# New Microbicidal Functions of Tracheal Glands: Defective Anti-Infectious Response to *Pseudomonas aeruginosa* in Cystic Fibrosis

**DOI:** 10.1371/journal.pone.0005357

**Published:** 2009-04-28

**Authors:** Sonia Bastonero, Yannick Le Priol, Martine Armand, Christophe S. Bernard, Martine Reynaud-Gaubert, Daniel Olive, Daniel Parzy, Sophie de Bentzmann, Christian Capo, Jean-Louis Mege

**Affiliations:** 1 Unité de Recherche sur les Maladies Infectieuses Tropicales et Emergentes, CNRS UMR 6236, Faculté de Médecine, Marseille, France; 2 Transcriptomic platform, Institut de Médecine Tropicale du Service de Santé des Armées, Marseille, France; 3 UMR Nutriments Lipidiques et Prévention des Maladies Métaboliques, INSERM U476 INRA UMR1260, Faculté de Médecine, Marseille, France; 4 Laboratoire d'Ingénierie des Systèmes Macromoléculaires, CNRS-IMM-UPR 9027, Marseille, France; 5 Fédération des Maladies Respiratoires, Hôpital de Sainte Marguerite, Marseille, France; 6 Institut Paoli Calmettes, INSERM Unité 891, Centre de Recherche en Cancérologie, Marseille, France; LMU University of Munich, Germany

## Abstract

Tracheal glands (TG) may play a specific role in the pathogenesis of cystic fibrosis (CF), a disease due to mutations in the *cftr* gene and characterized by airway inflammation and *Pseudomonas aeruginosa* infection. We compared the gene expression of wild-type TG cells and TG cells with the *cftr* ΔF508 mutation (CF-TG cells) using microarrays covering the whole human genome. In the absence of infection, CF-TG cells constitutively exhibited an inflammatory signature, including genes that encode molecules such as IL-1α, IL-β, IL-32, TNFSF14, LIF, CXCL1 and PLAU. In response to *P. aeruginosa*, genes associated with IFN-γ response to infection (CXCL10, IL-24, IFNγR2) and other mediators of anti-infectious responses (CSF2, MMP1, MMP3, TLR2, S100 calcium-binding proteins A) were markedly up-regulated in wild-type TG cells. This microbicidal signature was silent in CF-TG cells. The deficiency of genes associated with IFN-γ response was accompanied by the defective membrane expression of IFNγR2 and altered response of CF-TG cells to exogenous IFN-γ. In addition, CF-TG cells were unable to secrete CXCL10, IL-24 and S100A8/S100A9 in response to *P. aeruginosa*. The differences between wild-type TG and CF-TG cells were due to the *cftr* mutation since gene expression was similar in wild-type TG cells and CF-TG cells transfected with a plasmid containing a functional *cftr* gene. Finally, we reported an altered sphingolipid metabolism in CF-TG cells, which may account for their inflammatory signature. This first comprehensive analysis of gene expression in TG cells proposes a protective role of wild-type TG against airborne pathogens and reveals an original program in which anti-infectious response was deficient in TG cells with a *cftr* mutation. This defective response may explain why host response does not contribute to protection against *P. aeruginosa* in CF.

## Introduction

Cystic fibrosis (CF), the most common fatal hereditary disease in the Caucasian population, is due to mutations in the Cystic Fibrosis Transmembrane conductance Regulator (*cftr*) gene. Over 1500 mutations have been described and the most frequent of these is a phenylalanine deletion in position 508 (ΔF508) (http://www.genet.sickkids.on.ca/cftr mutations). CF is characterized by exocrine gland alteration in multiple organs, but its major manifestation is chronic lung disease due to airway inflammation and bacterial colonization [1]. The precise relationship between *cftr* mutations and the development of lung disease has yet to be determined. The lungs of CF newborns exhibit abnormal mucus secretion [2], and early inflammatory responses are present in the airways of CF fetuses and newborns [3], [4]. The excessive production of interleukin (IL)-1, IL-6 and IL-8 by epithelial cells with a mutated *cftr* gene [5]–[7] also suggests that an unrestricted inflammatory reaction occurs in the airways of CF patients. This excessive inflammatory reaction is not protective against opportunistic pathogens such as *Pseudomonas aeruginosa*, a gram-negative bacterium that usually does not infect the lungs of healthy individuals, but is largely responsible for airway obstruction and the decline of pulmonary function in CF patients [8], [9]. It has been found that a large arsenal of products secreted by *P. aeruginosa* contributes to persistent infection of the lungs of CF patients and subsequent lung lesions [9].

Although their role is less known than that of epithelial cells [10], tracheal glands (TG) may play a critical role in the pathophysiology of CF [11]. TG cells express high levels of CFTR as compared with other bronchial epithelial cell types [12], and secrete a wide variety of proteins including mucins, antibacterial molecules, cytokines, chemokines and lipid mediators [13]–[16]. These properties would confer a role to TG in lung homeostasis as already described for epithelial cells and in lung defense against infection [17]. However, the role of TG with a functional *cftr* gene or the *cftr* ΔF508 mutation (CF-TG) in pathogen persistence has not been investigated. The elevated viscosity of TG fluid may be an important factor in promoting airway disease and bacterial colonization [18]. Here, we took advantage of the existence of TG and CF-TG cell lines [19], [20] to compare their gene expression profiles using microarrays covering the entire human genome. Soluble products released by a *P. aeruginosa* strain isolated from a CF patient stimulated the expression of genes critically involved in host defense in TG cells but not in CF-TG cells. This deficiency was related to the impairment of the IFN-γ pathway, which is essential for host microbicidal response. Hence, the inability of CF-TG cells to activate genes with microbicidal competence may explain why host response does not contribute to protection against *P. aeruginosa* in cystic fibrosis.

## Results

### Transcriptional Profile of CF-TG Cells

**Figure 1:**
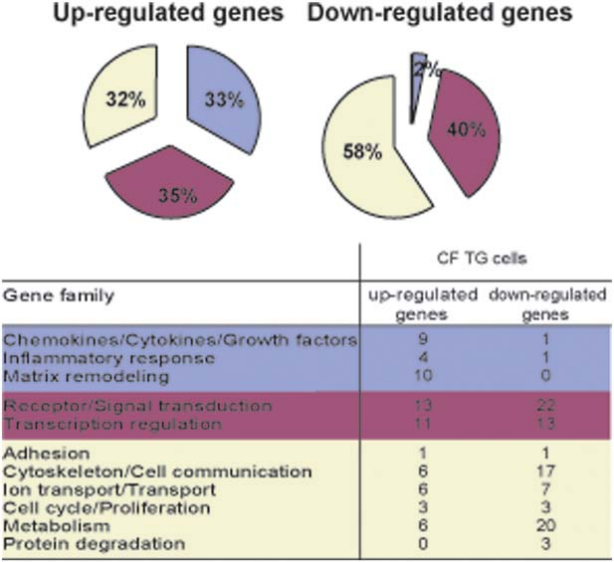
Functional categories of genes in CF-TG cells. The genes expressed by wt TG and CF-TG cells were analyzed by microarrays. Up-regulated and down-regulated genes in CF-TG cells compared to wt TG cells were classified in families according to their known function. The major biological processes are represented by wedges, including the percentage of 69 up-regulated and 88 down-regulated genes.

We analyzed the genes modulated in CF-TG cells compared to wild-type (wt) TG cells by whole-genome microarrays. We found that 157 genes were significantly modulated consisting of 69 up-regulated genes and 88 down-regulated genes. The 69 up-regulated genes in CF-TG cells (see Table S1 for the entire microarray database) were classified by families according to their known function (Figure 1). They are distributed in three groups of genes. Thirty-three percent of genes (23 genes) belong to families involved in the innate immune response (chemokines/cytokines/growth factors, inflammatory response, matrix remodeling). They included IL-1α, IL-1β, IL-32, TNF ligand superfamily member 14 (TNFSF14 also known as LIGHT), leukemia inhibitory factor (LIF), CXCL1 (Gro-α) and plasminogen activator urokinase (PLAU) (Table S1). Thirty-five percent of genes belong to the receptor/signal transduction and transcription regulation families and 32% of genes encoded proteins involved in unrelated cell functions. Note that most of the up-regulated genes in CF-TG cells exhibited a fold change (FC) lower than 3.0 (52/69 genes, Table S1). The 88 down-regulated genes in CF-TG cells compared to wt TG cells (see Table S2) belong to the receptor/signal transduction and transcription regulation families (40% of the total number of down-regulated genes) while 58% of these genes encoded molecules involved in adhesion, cytoskeletal architecture, cell communication, transport and ion transport, cell cycle, proliferation, metabolism and protein degradation. Only two genes involved in the innate immune response were down-regulated (Figure 1). These results showed that the transcriptional profile of CF-TG cells was moderately inflammatory compared to that of wt TG cells.

### Proteins Released by *P. aeruginosa* strains

**Figure 2:**
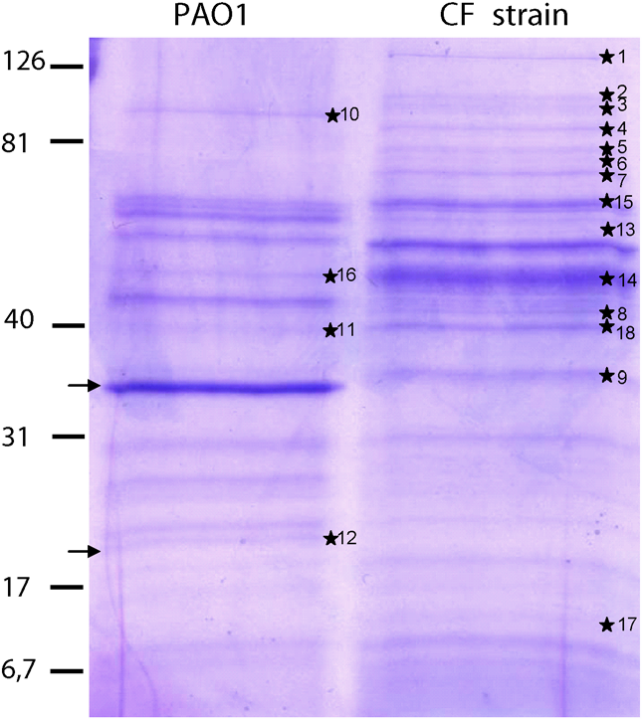
Analysis of protein content of the PAO1 and the CF strain supernatants. Proteins secreted by *P. aeruginosa* after a 18 h-culture (*) were cut out from Coomassie blue stained gels and subjected to mass spectrometry analysis using MALDI-TOF. Arrows designate the LasB protein-derivatives.

The bacterial strain isolated from one CF patient and the reference PAO1 strain were cultured overnight, and the extracellular products were analyzed by electrophoresis. The two protein profiles were markedly different (Figure 2). Some proteins were released only by the PAO1 strain (Table 1). This is the case for elastase, LasB, (upper arrow, Figure 2), the Type 2 secretion system (T2SS) Xcp classical substrate that is the major secreted protein and band 12 (lower arrow, Figure 2), which corresponds to the peptide (about 20 kDa) cleaved from the proenzyme LasB before activation. Other proteins, including OprF (bands 11 and 18) and a probable T2SS substrate since it is absent in a Δ*xcp*Δ*xqh* mutant [21], result confirmed by one of us (G. Michel in SdB's team, personal unpublished data) (bands 3 and 10), were released by both strains (Table 1). Finally, different proteins were released only by the CF strain. They included bands 1, 2, 4–9, 13, 14 (surprisingly, FliC was only detected in extracellular products of the CF strain), 15 and 17 (Table 1). The band 17 corresponds to the Hcp1 protein of the T6SS that has been detected in sputum of CF patients [22]. The band 9 (PstS extracellular phosphate-binding protein), recently described to form PstS-rich appendages through the T2SS HxC apparatus, is induced during phosphate limitation, thus playing a key role in *P. aeruginosa* virulence assessed in a murine model [23]. Bands 6–8 and band 13, a mixture of at least two proteins, are classically produced under phosphate limitation as PstS. This protein pattern suggests that the phosphate limitation-like secretome of the CF bacterial strain is remarkedly stable even after bacterial culture. As the CF bacterial strain is more relevant than the reference strain for investigation of glandular response in the context of CF, we performed further experiments with extracellular products from the clinical *P. aeruginosa* CF strain.

**Table 1:** Secretomes of the *P. aeruginosa* strains.

Numbers on gel	PA	Name	Function	MW (kDa)	PAO1 strain	CF strain
**1**	PA4270	DNA-directed RNA polymerase beta chain		151	−	++
**2**	ND	ND	ND	110	−	++
**3 and 10**	PA0572	hypothetical protein	unknown function probable T2SS substrate	100	+	++
**4**	PA4266	Elongation factor G		78	−	++
**5**	PA4761	DnaK protein		68	−	++
**6**	PA3319	PlcN	non-hemolytic phospholipase C precursor	77	−	++
**7**	PA2635	Hypothetical protein	PhoX, predicted phosphatase	74	−	++
**8**	PA0347	GlpQ	glycerophosphoryl diester phosphodiesterase, periplasmic	42	−	++
**9**	PA5369	PstS	phosphate ABC transporter, periplasmic phosphate-binding protein, PstS	34	−	++
**10 and 3**	PA0572	hypothetical protein	unknown function probable T2SS substrate	100	+	++
**11 and 18**	PA1777	OprF	major porin and structural outer membrane OprF precursor	38	+	++
**12**	PA3724	LasB	prepeptide of LasB	21	+	−
**13**	PA3770	GuaB	inosine-5′-monophosphate dehydrogenase	52	−	+
**13**	PA3910	hypothetical protein	phosphodiesterase/alkaline phosphatase D	59	−	+
**14**	PA1092	FliC	flagellin type B	49	−	+++
**15**	PA4385	GroEL	chaperones & heat shock proteins	57	−	+
**16**	PA2623	Icd	isocitrate dehydrogenase	46	+	−
**17**	PA0085	Hcp1	type VI secretion system effector, Hcp1 family	17	−	+
**18 and 11**	PA1777	OprF	major porin and structural outer membrane OprF precursor	38	+	++

Proteins secreted by the *P. aeruginosa* strain isolated from the patient with CF (CF strain) and the PAO1 strain were cut out from Coomassie blue stained gels and subjected to mass spectrometry analysis using MALDI-TOF. PA is the gene number on genome annotation available at http://www.pseudomonas.com. ND: not determined due to undetectable ions.

### Transcriptional Profile of wt TG and CF-TG Cells in Response to *P. aeruginosa*

**Figure 3:**
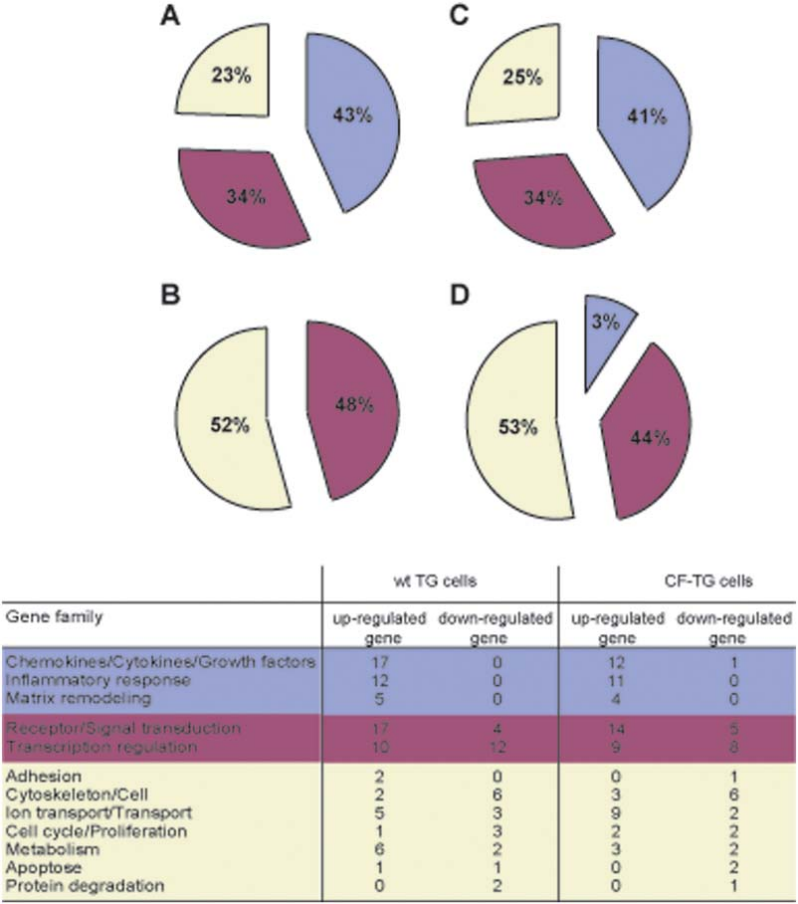
Functional categories of genes in stimulated TG cells. The genes expressed by wt TG and CF-TG cells stimulated with *P. aeruginosa*-secreted products were analyzed by microarrays. Up-regulated and down-regulated genes were classified in families according to their known function. The major biological processes are represented by wedges, including the percentage of modulated genes. Seventy eight genes were up-regulated (A) and 33 genes were down-regulated (B) in stimulated wt TG cells; 67 genes were up-regulated (C) and 30 genes were down-regulated (D) in stimulated CF-TG cells.

We wondered whether *P. aeruginosa*-secreted products affected the transcriptional response of wt TG cells and CF-TG cells. First, *P. aeruginosa*-secreted products significantly up-regulated the expression of 78 genes (see Table S3, and Figure 3) in wt TG cells compared to unstimulated wt TG cells. For the majority of these genes (52), the FC value was higher than 3.0 and could reach a 50-fold increase (Table S3). Forty-three percent of the up-regulated genes encode proteins involved in the innate immune response, 34% of genes belong to receptor/signal transduction and transcription regulation families, and 23% of genes encode proteins involved in other biological processes (Figure 3A). *P. aeruginosa*-secreted products moderately inhibited the expression of 33 genes by 1.5 to 3.0 fold (see Table S4). Among them, 48% of genes belong to the receptor/transduction signal and transcription regulation families and 52% of genes encode proteins involved in adhesion, cytoskeletal architecture, cell communication, transport, cell cycle, proliferation, metabolism, apoptosis and protein degradation. No gene belonging to the innate immune response family was down-modulated (Table S4 and Figure 3B).

Second, *P. aeruginosa*-secreted products also affected the transcriptional response of CF-TG cells compared to unstimulated CF-TG cells. The number of modulated genes (97) was similar to that found in stimulated TG cells (111) but the level of gene modulation was lower. Indeed, among 67 genes significantly up-regulated in CF-TG cells, the FC value did not exceed 8 fold and was lower than 3.0 for the majority of them (52/67) (Table S5). The distribution of these genes in functional families was similar to that of TG cells stimulated with *P. aeruginosa* products (Figure 3C): 41% of them encode proteins involved in the innate immune response, 34% belong to receptor/signal transduction and transcription regulation families, and 25% of genes encode molecules involved in other cell functions. *P. aeruginosa*-secreted products inhibited the expression of 30 genes in CF-TG cells (Table S6); most of these genes exhibited a FC comprised between 1.5 and 3.0. Only one inflammatory gene was down-modulated in *P. aeruginosa*-stimulated CF-TG cells. As found in wt TG cells stimulated with *P. aeruginosa* products, 44% of genes belong to receptor/transduction signal and transcription regulation families, and 53% of genes encode proteins involved in other cell functions (Figure 3D).

### Analysis of Modulated Genes in *P. aeruginosa*-Stimulated Cells

**Figure 4:**
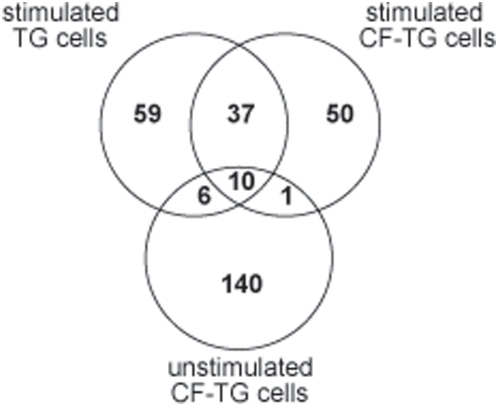
Venn diagram of differentially gene expression. A total of 157 modulated genes in CF-TG cells, 111 in wt TG cells stimulated with *P. aeruginosa*-secreted products and 98 in CF-TG cells stimulated with *P. aeruginosa* products were compared. Intersection showed 46 genes modulated within wt TG and CF-TG cells stimulated with *P. aeruginosa*-secreted products, and 10 genes were common in the three groups of cells.

**Figure 5:**
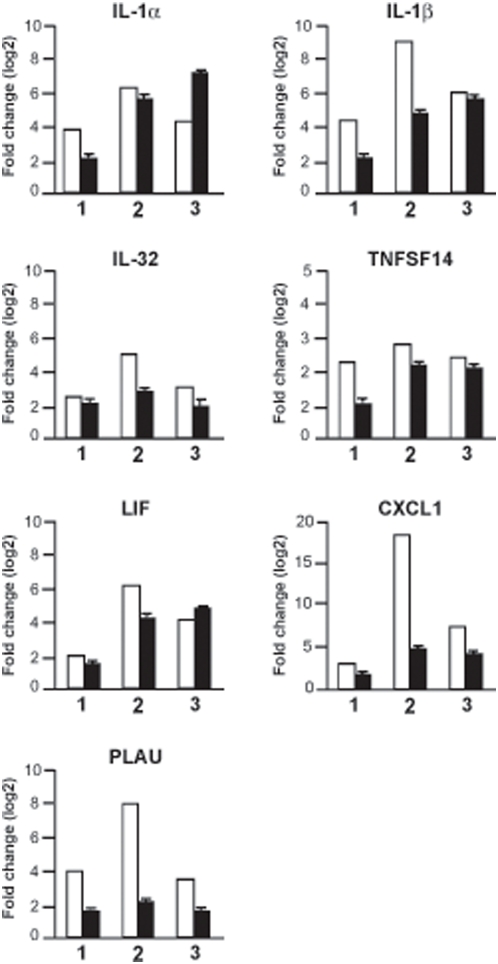
Validation of seven target sequences using qRT-PCR. The expression levels of seven genes commonly regulated by CF-TG cells and wt TG cells stimulated with *P. aeruginosa*-secreted products were determined by microarray (white bar) and qRT-PCR (black bar). The results of microarrays are expressed as the log2 ratio of the fold change, and the results of qRT-PCR represent means±SEM of three experiments performed in triplicate. (1) unstimulated CF-TG cells vs. unstimulated wt TG cells; (2) stimulated vs. unstimulated wt TG cells; (3) stimulated vs. unstimulated CF-TG cells.

**Figure 6:**
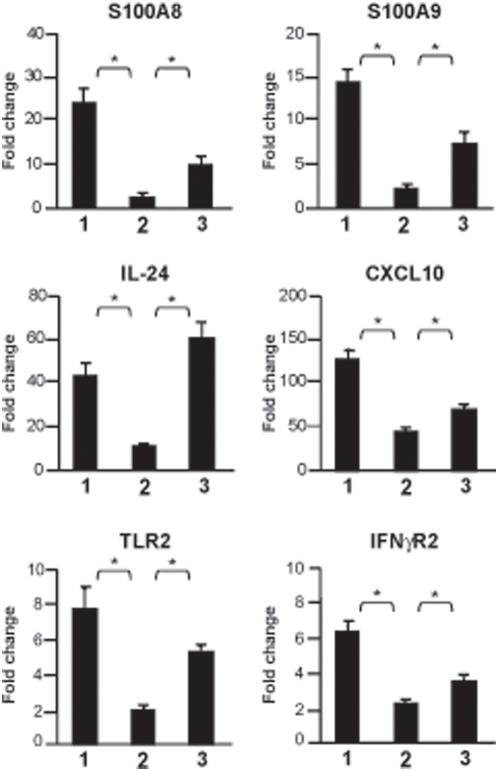
Impaired immune response in *P. aeruginosa*-stimulated CF-TG cells. The expression levels of six genes involved in the innate immune response were studied by qRT-PCR. Results, which represent means±SEM of three experiments performed in triplicate, are expressed as fold change. (1) stimulated vs. unstimulated wt TG cells; (2) stimulated vs. unstimulated CF-TG cells; (3) stimulated vs. unstimulated *cftr*-transfected CF-TG cells. *P<0.05.

Since the distribution of genes was similar in TG and CF-TG cells stimulated with *P. aeruginosa*-secreted products, we wondered which genes were commonly or specifically modulated. Using a Venn diagram (Figure 4), we first reported an inflammatory signature consisting of 46 genes commonly modulated in both stimulated cell lines (grayed in tables S3, S4, S5, S6). Among them, seven genes were also up-regulated in unstimulated CF-TG cells (grayed in Table S1). The expression of these genes, namely those encoding IL-1α, IL-1β, IL-32, TNFSF14, LIF, CXCL1 and PLAU, was validated by real time RT-PCR. The fold changes found in microarray and real-time PCR experiments related with each other (Figure 5). Second, 59 genes were modulated in wt TG cells stimulated with *P. aeruginosa*-secreted products, but not in stimulated CF-TG cells (Figure 4). They included 17 genes involved in the innate immune response such as those that encode CXCL10, which was up-regulated by 49 fold, granulocyte-macrophage colony stimulating factor (CSF2), IL-24, matrix metalloproteinases (MMP1 and MMP3), IFN-γ receptor (IFNγR2) and toll-like receptor (TLR2). We called this group of genes microbicidal signature. The expression of three genes encoding S100 calcium-binding proteins A was also up-regulated. Real time RT-PCR confirmed the data obtained with the microarray approach. *P. aeruginosa*-secreted products strongly induced the expression of the genes encoding CXCL10, IL-24, TLR2, IFNγR2 and S100A8 in wt TG cells, but weakly in CF-TG cells. The differences between the FC found in wt TG cells and CF-TG cells were significant (*P*<0.05) (Figure 6, compare lines 1 and 2). Since S100A8 forms a heterodimer with S100A9 (calprotectin) [24], we also studied the expression of the gene encoding S100A9. This gene was significantly (*P*<0.05) higher in wt TG cells stimulated with *P. aeruginosa* products than in stimulated CF-TG cells (Figure 6). Third, 49 genes were modulated in CF-TG cells stimulated with *P. aeruginosa* products but were unaffected in stimulated wt TG cells (Figure 4). Among them, 11 were involved in the innate immune response. They included the genes that encoded IL-8, CXCL2, IL-1 receptor antagonist (IL1RN), a powerful immunoregulatory molecule, lipocalin (LCN2) and serum amyloid (SAA1 and SAA2) (compare Tables S5 and S3). Taken together, these results showed that CF-TG cells stimulated with *P. aeruginosa* products exhibited a specific transcriptional program in which microbicidal signature was defective.

### Transcriptional Program and *cftr* Mutation

We wondered if the transfection of CF-TG cells with a plasmid containing a functional *cftr* gene induced a transcriptional pattern similar to that of wt TG cells. First, we assessed the expression of genes encoding IL-1α, IL-1β, IL-32, TNFSF14, LIF, CXCL1 and PLAU, which represented the constitutive inflammatory signature of CF-TG cells, by real-time quantitative RT-PCR (qRT-PCR). We clearly demonstrated that the *cftr* gene transfer corrected the constitutive inflammation of CF-TG cells. Indeed, the ratio of the gene expression levels of *cftr*-transfected CF-TG cells and wt TG cells was near 1, while it was significantly increased in CF-TG cells compared to wt TG cells (Table 2). In contrast, the transfection of CF-TG cells with a control plasmid did not affect the gene expression of CF-TG cells (data not shown). Second, we selected 6 genes from the microbicidal signature that were up-regulated in wt TG cells stimulated with *P. aeruginosa* products but not in stimulated CF-TG cells, namely the genes that encode CXCL10, IL-24, TLR2, IFNγR2, S100A8 and S100A9. When CF-TG cells were transfected with a functional *cftr* gene and stimulated with *P. aeruginosa*-secreted products, they expressed the transcriptional signature characteristics of wt TG cells stimulated with *P. aeruginosa* products. Indeed, the FC of the genes encoding CXCL10, IL-24, IFNγR2, TLR2, S100A8 and S100A9 were significantly (*P*<0.05) higher in *cftr*-transfected CF-TG cells than in CF-TG cells, and were near of those of the genes expressed by wt TG cells stimulated with *P. aeruginosa* products (Figure 6). Again, the transfection of CF-TG cells with a control plasmid did not modify the gene expression of CF-TG cells in response to *P. aeruginosa* products (data not shown). Taken together, these results demonstrated that the constitutive inflammatory program and the defective microbicidal signature of CF-TG cells were related to the *cftr* ΔF508 mutation.

**Table 2:** Inflammatory program of CF-TG cells transfected with *cftr* gene.

Gene	fold change	
	CF-TG cells/wt TG cells	*cftr*-transfected CF-TG cells/wt TG cells
IL-1α	4.3±0.4	1.2±0.1*
IL-1β	4.5±0.7	1.6±0.3*
IL-32	4.6±0.7	1.7±0.4*
TNFSF14	2.1±0.2	1.7±0.1
LIF	2.7±0.2	0.8±0.1*
CXCL1	3.7±0.2	0.7±0.05*
PLAU	2.9±0.1	0.8±0.1*

The expression levels of seven commonly regulated genes were determined by qRT-PCR. The results are expressed as the fold changes between CF-TG cells and wt TG cells, and between CF-TG cells transfected with *cftr* gene and wt TG cells. They represent means±SEM of three experiments.

* *P*<0.05.

### Functional Analyses of Inflammatory and Microbicidal Signatures of CF-TG cells

Since the expression of the genes that encode IL-1α, IL-β, LIF and CXCL1 was higher in CF-TG cells when compared to that of wt TG cells, we wondered whether the spontaneous release of these inflammatory mediators after 24 h of culture was also increased in CF-TG cells. The cytokine content of cell supernatants was determined by ELISA. The spontaneous release of IL-1α, IL-β, LIF and CXCL1 was significantly (*P*<0.05) increased in CF-TG cells (Table 3), demonstrating that the transcriptional inflammatory signature of CG-TG cells corresponded to a functional signature.

**Table 3:** Release of inflammatory cytokines by TG cells.

Cytokines	wt TG cells	CF-TG cells
IL-1α	1975±125	4200±150*
IL-1β	0.035±0.015	2.5±0.15*
LIF	0	45±6.5*
CXCL1	4200±85	7500±375*

The spontaneous release of inflammatory cytokines by wt TG cells and CF-TG cells was determined by ELISA. The results are expressed in pg per 2×10^6^ cells. They represent means±SEM of three experiments.

* *P*<0.05.

**Figure 7:**
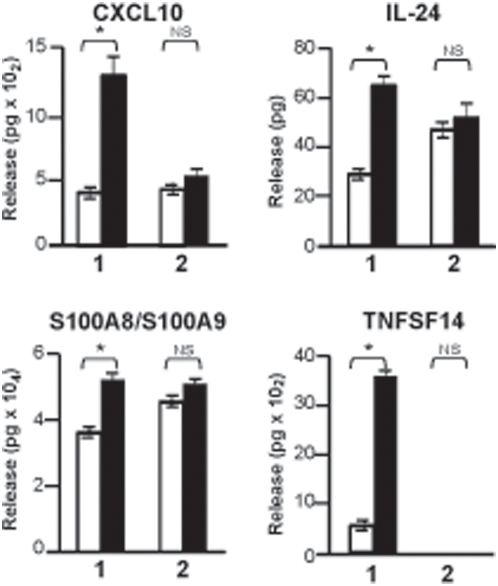
Release of cytokines of the innate immune response by TG cells. Wild type TG and CF-TG cells (2×10^6^ cells per assay) were incubated in the absence (white bar) or presence (black bar) of *P. aeruginosa*-secreted products for 24 h. The release of cytokines of the innate immune response by wt TG cells (1) and CF-TG cells (2) was determined by ELISA. The results are expressed in pg of cytokines. They represent means±SEM of three experiments. *P<0.05. NS: non significant.

**Figure 8:**
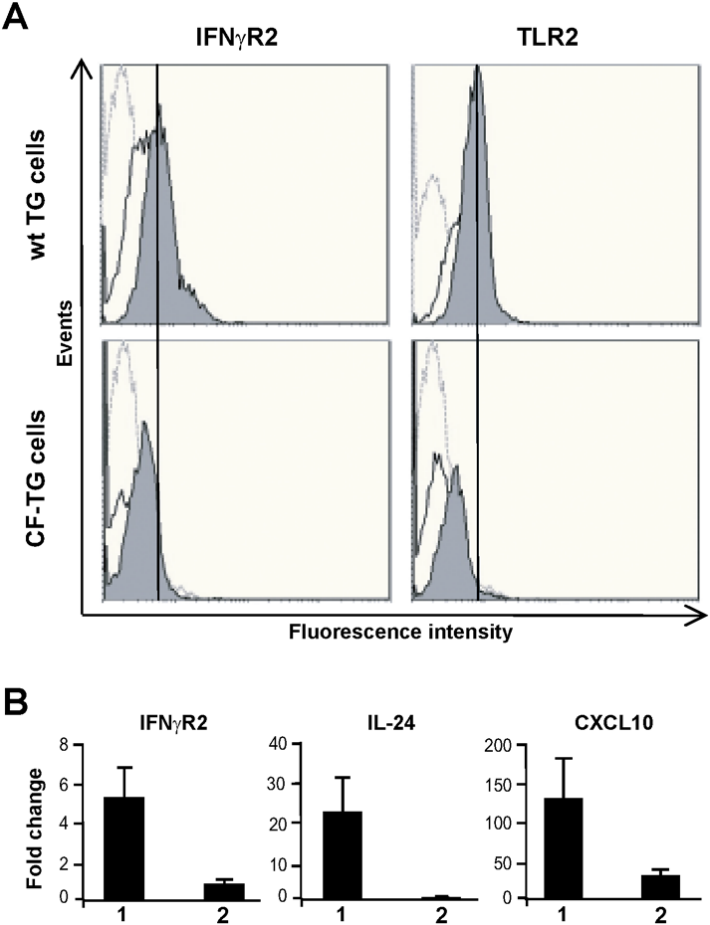
Receptor expression, and effect of IFNγ on gene expression in CF-TG cells. A, wt TG and CF-TG cells were incubated in the absence or presence of *P. aeruginosa*-secreted products for 24 h. The membrane expression of IFNγR2 and TLR2 was determined by flow cytometry. Histograms are representative of three different experiments. Hatched line, control IgG; Full line, unstimulated cells; Grey color, cells stimulated with *P. aeruginosa*-secreted products. B, 1000 UI of IFNγ were added to wt TG cells (1) and CF-TG cells (2) for 3 h, and the expression of genes encoding IFNγR2, Il-24 and CXCL10 was studied by qRT-PCR. Results, which represent means±SEM of three experiments, are expressed as fold change. (1) IFNγ-treated vs. untreated wt TG cells; (2) IFNγ-treated vs. untreated CF-TG cells.

Next, we tempted to relate the silencing microbicidal signature in CF-TG cells with the expression of effectors. First, we studied the release of CXCL10, IL-24, S100A8/S100A9 and TNFSF14 by wt TG cells and CF-TG cells stimulated with *P. aeruginosa*-secreted products by ELISA. *P. aeruginosa* products were unable to stimulate the release of CXCL10, IL-24, S100A8/S100A9 and TNFSF14 by CF-TG cells but clearly stimulated their secretion by wt TG cells (Figure 7). This inability of CF-TG cells to produce cytokines in response to *P. aeruginosa*-secreted products was specific of cytokines involved in microbicidal response. Indeed, the release of IL-1α and LIF was significantly increased (*P*<0.05) in CF-TG cells stimulated with *P. aeruginosa* products compared to resting CF-TG cells (4950±50 vs. 4200±140 pg/2×10^6^ cells for IL-1α; 425±42 vs. 45±7 pg/2×10^6^ cells for LIF). Second, the membrane expression of IFNγR2 and TLR2 proteins by wt and CF-TG cells was studied by flow cytometry. Clearly, the membrane expression of IFNγR2 and TLR2 was lower in CF-TG cells stimulated with *P. aeruginosa*-secreted products than in stimulated wt TG cells (Figure 8A). The defective expression of IFNγR2 had functional consequences. Indeed, the treatment of wt TG cells with human recombinant IFNγ resulted in the increased expression of genes encoding IFNγR2, IL-24 and CXCL10. In contrast, IFNγ was unable to stimulate their expression in CF-TG cells (Figure 8B), suggesting that CF-TG cells were unable to mount a microbicidal program even in response to IFNγ.

### Altered Sphingolipid Pathway in CF-TG cells

**Figure 9:**
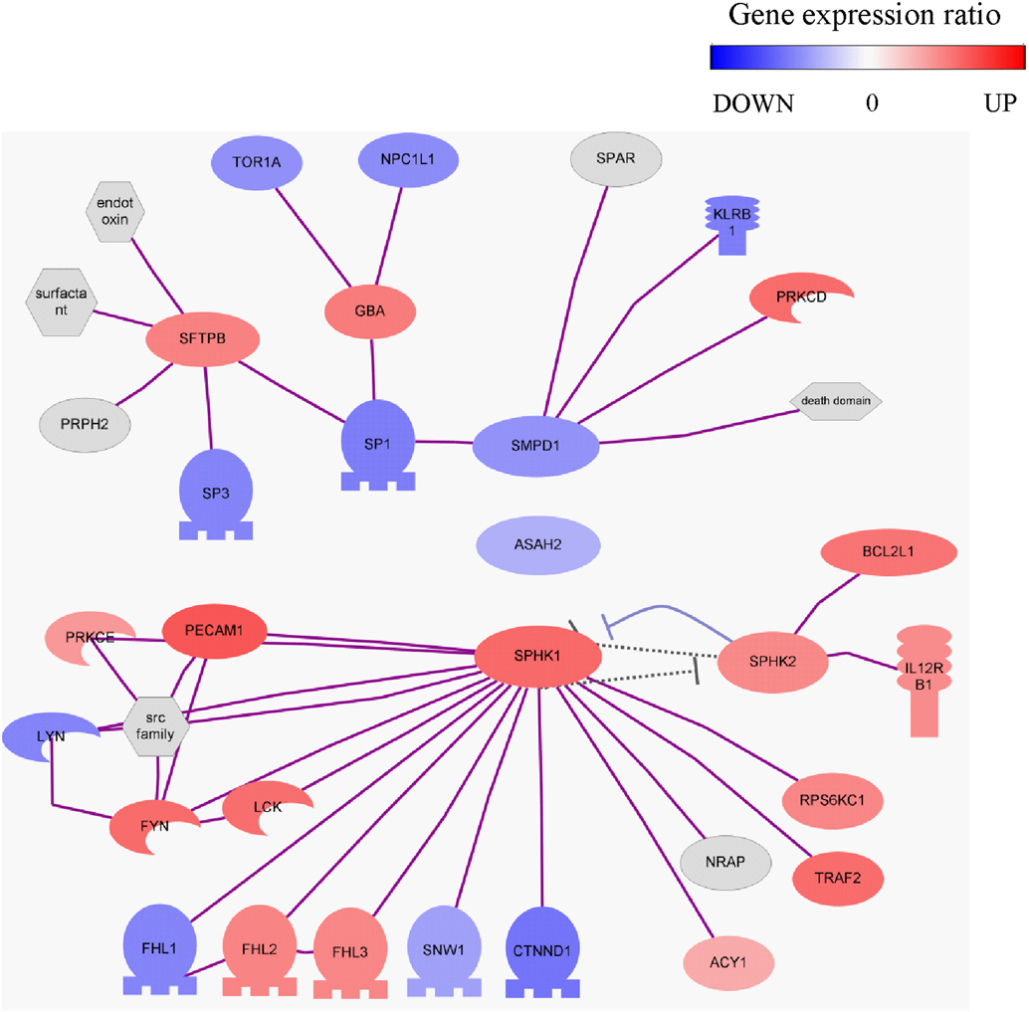
Analysis of genes of the sphyngolipid pathway in wt TG and CF-TG cells. Microarray data were collected, and the network of genes encoding enzymes involved in the sphyngolipid metabolism and predicted target genes that are significantly modulated was represented. Red color corresponded to up-regulated genes in CF-TG cells compared to wt TG cells and blue color to down-regulated genes.

**Figure 10:**
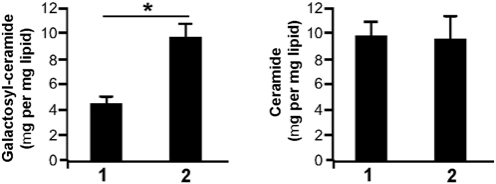
Ceramide levels in wt TG cells and CF-TG cells. Total lipids were extracted from wt TG cells (1) and CF-TG cells (2), and separated by one-dimensional thin-layer chromatography. Galactosyl-ceramides and ceramides were quantified by scanning using standard calibration curves (r2). The results, which represent means±SEM of 5 determinations, are expressed in µg galactosyl-ceramide (A) or ceramide (B) per mg of total lipids. *P<0.05.

**Figure 11:**
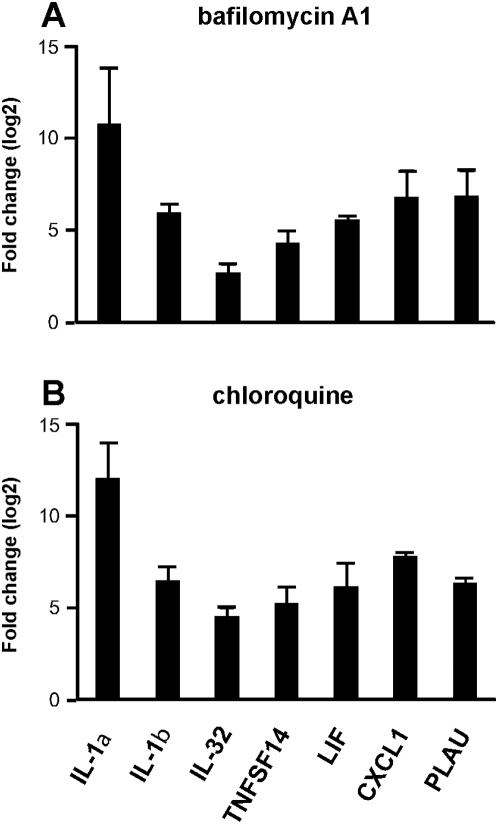
Role of alkalinization on the inflammatory signature of wt TG cells. Wild type TG cells were treated with 300 nM bafilomycin A1 (A) or 200 µM chloroquine (B) for 3 h. The expression levels of the seven genes involved in the inflammatory signature of CF-TG cells were studied by qRT-PCR. Results are expressed as the log2 ratio of the fold change observed in the presence of alkalinization agent vs. in its absence. They represent the means±SEM of three experiments.

It has been recently shown that altered metabolism of sphingolipids due to defective acidification of intracellular vesicles might account for constitutive inflammation of lungs from *cftr*-deficient mice [25]. We wondered if such mechanism was found in CF-TG cells and may account for their inflammatory signature. First, the analysis of microarray data suggests an imbalance in sphingolipid metabolism. It revealed that the expression of galactosyl-ceramidase was down-regulated in CF-TG cells compared to wt TG cells (Table S2). Several key components of the ceramide metabolism are represented in the network of modulated genes in CF-TG cells (Figure 9). They included the enzymes involved in the sphingomyelin degradation pathway, i.e. sphyngomyelin phosphodiesterase 1 (SMPD1), N-acylsphingosine amidohydrolase 2 (ASAH2), sphingosine kinase (SPHK) 1 and 2. Interestingly, SMPD1, ASAH2 and downstream genes were down-modulated in CF-TG cells as compared to wt TG cells. In contrast, genes encoding SPHK1 and related genes were up-regulated. Note that several transcription factors, including SP1, SP3 and CTNND1, were down-regulated in CF-TG cells compared to wt TG cells and that the SFTPB gene encoding the SP-B protein involved in the reduction of pulmonary surfactant properties [26] was up-regulated in CF-TG cells. Second, sphingolipids were quantified in wt TG and CF-TG cells. Galactosyl-ceramides (galactocerebrosides) were significantly (*P*<0.01) increased in CF-TG cells as compared to wt TG cells while ceramide levels were similar in CF-TG cells and wt TG cells (Figure 10). Third, wt TG cells were treated with bafilomycin A1 and chloroquine to alkalinize intracellular vesicles, and the inflammatory signature of wt TG cells was determined. Bafilomycin A1 used at 300 nM significantly increased the expression of genes encoding IL-1α, IL-1β, IL-32, TNFSF14, LIF, CXCL1 and PLAU (Figure 11A). Chloroquine at 200 µM also increased the expression of the inflammatory signature of wt TG cells (Figure 11B). Doses of 30 nM of bafilomycin A1 and 20 µM of chloroquine did not affect the expression of inflammatory genes in wt TG cells (data not shown). Taken together, these results suggest that altered metabolism of sphingolipids and defective acidification of intracellular organelles may account for the inflammatory signature of CF-TG cells.

## Discussion

We wondered whether TG may play a role in airway defense against *P. aeruginosa* since they are known to secrete a wide variety of molecules involved in bacterial defense [13]–[16] and that the increased viscosity of TG fluid in CF may favor bacterial infection [18]. We first selected a *P. aeruginosa* strain, which was the first strain from clone A lineage isolated from a ΔF508 homozygous patient. As bacteria do not reach the bronchial epithelium but rather stick in the viscous mucus overlaying the epithelium [27], [28], we chose to use extracellular products released by *P. aeruginosa* but not viable bacteria. It is likely that these released products reach glands from the epithelium surface since these two compartments are connected through connecting tubes. In addition, it has been demonstrated that the rhinovirus infection of submucosal gland cells induces mucin production [29]. Consequently, we stimulated TG cells with products secreted by a strain of *P. aeruginosa* isolated from a patient with CF.

We found that *P. aeruginosa*-secreted products stimulated a microbicidal response in wt TG cells dependent, at least in part, on the IFN-γ pathway. First, the expression of genes encoding mediators such as CXCL10, IL-24, TLR2, IFNγR2 and S100 calcium-binding proteins A was up-regulated. CXCL10 (interferon-gamma-inducible protein 10, IP10) is a target of the IFN-γ pathway [30]. IL-24 is able to stimulate IFN-γ production by mononuclear cells besides its major role in wound healing and cell proliferation [31]. Second, the expression of the receptor of IFN-γ (IFNγR2) by wt TG cells was increased in response to *P. aeruginosa*-secreted products. Third, adding IFNγ to wt TG cells up-regulated the expression of genes encoding IFNγR2, IL-24 and CXCL-10, suggesting that the IFNγ pathway plays a major role in the microbicidal response of wt TG cells to *P. aeruginosa* products. The increased membrane expression of TLR2 by wt TG cells in response to *P. aeruginosa*-secreted products may also participate to innate immune response. It has been demonstrated that TLR2 recognizes *P. aeruginosa* [32] and that this pathogen activates airway epithelial cells through recognition by TLR2 [33]. The expression of S100 calcium-binding proteins A8 (Mrp8) and A9 (Mrp14) was also markedly up-regulated in TG cells. These proteins display potent antimicrobial activity [24], [34], and are expressed in bronchial epithelial cells exposed to *P. aeruginosa* [35]. S100A8 and S100A9 also interact with TLR4 to amplify phagocyte activation [36], and are under the control of IL-10 family members [37]. Finally *P. aeruginosa*-secreted products dramatically increased the release of TNFSF14 by wt TG cells. TNFSF14 is known to be expressed by T lymphocytes [38] and dendritic cells [39]. TNFSF14 that belongs to the TNF superfamily is involved in inflammation and immune response through its role on IFN-γ [40] and IL-12 [41] production. To our knowledge, this is the first demonstration that TNFSF14 is secreted by wt TG cells.

We also found that numerous genes including inflammatory genes were constitutively up-regulated in CF-TG cells compared to wt TG cells. The moderate inflammatory signature of CF-TG cells consisted of inflammatory cytokines such as IL-1 family members and CXCL1 (Gro-α), known to be associated with CF [42], [43], and inflammatory genes not previously associated with the disease such as IL-32, TNFSF14, LIF and PLAU. IL-32, a cytokine recently described, induces the expression of TNF, IL-1, IL-6 and chemokines [44], [45]. LIF, which may act as a pro- or anti-inflammatory cytokine, has an important role in chronic airway inflammation [46] and stimulates the production of chemotactic factors by monocytes [47]. PLAU is involved in inflammation and matrix remodeling with ADAM8 or SERPIN, and might induce gland hyperplasia [48]. We can suppose that the up-regulation of PLAU or associated genes is related to the hyperplasia found in CF airways [49]. Note that a potential secreted virulence factor from *P. aeruginosa* degrades PLAU receptor [50], suggesting that the PLAU pathway is critical in host response to bacterial aggression. The constitutive inflammatory signature observed in CF-TG cells was due to the *cftr* ΔF508 mutation since the transfection of CF-TG cells with a plasmid containing a functional *cftr* gene prevented the up-regulation of inflammatory genes. Our results are consistent with the hypothesis that early pulmonary inflammation is already present before any infection in CF lungs [3], [4], [51]. They also suggest that the role of tracheal glands is critical in the pathophysiology of CF. First, CF is characterized by altered exocrine glands and it is likely that TG cells express higher levels of CFTR than other bronchial epithelial cells [12]. Second, our results clearly showed that the *cftr* ΔF508 mutation is related to up-regulated expression of inflammatory genes in CF-TG cells. In airway epithelial cells from the trachea or bronchi of CF donors, it has been shown that the *cftr* ΔF508 mutation has a minimal effect on the expression profile of ≈ 22,000 genes: only 18 genes were up-regulated. They include nine genes of unknown function and eight genes involved in transcription regulation, adhesion, cytoskeleton/cell communication, ion transport/transport and metabolism. Only one gene, which encodes a novel interleukin receptor, may be included in the inflammatory family [52].

Importantly, we demonstrated that *P. aeruginosa*-secreted products were unable to induce a microbicidal response in CF-TG cells in contrast to wt TG cells. Hence, genes from the IFN-γ pathway (IL-24, CXCL10 and IFNγR2) and other mediators of innate immune response such as TLR2, S100A8 and S100A9 were not modulated. The lack of IFN-γ signaling due to a mutation in the extracellular domain of IFNγR2 is associated with susceptibility to mycobacterial infection [53]. CF-TG cells also exhibited defective membrane expression of IFNγR2 after stimulation with *P. aeruginosa* products. Adding IFNγ to CF-TG cells did not stimulate the expression of genes from IFNγ pathway, in contrast to wt TG cells. The defective membrane expression of TLR2 by CF-TG cells may participate to decreased innate immune response. It is likely that the defective expression of TLR2 and the lack of calcium-binding proteins A combined with impaired IFN-γ pathway account for the inefficiency of the innate immune response toward *P. aeruginosa* in CF-TG cells. TNFSF14 may also be involved in defective response of CF-TG cells to *P. aeruginosa*-secreted products. Its blockade induces susceptibility of mice to *Leishmania major* through the down-modulation of IL-12 [41], suggesting that TNFSF14 may be a critical factor in the *P. aeruginosa* susceptibility of cells with a *cftr* gene mutation.

The differences in the constitutive inflammatory signature and the microbicidal responses induced by *P. aeruginosa* products between CF-TG cells and wt TG cells may be due to genetic and phenotypic differences since both cell lines have been isolated and established from two different donors. This was unlikely since we found that CF-TG cells exhibited an inflammatory pattern, as previously reported for CF epithelial cells [1], [3], [4]. Importantly, we clearly showed that the constitutive inflammatory signature of CF-TG cells and the impaired expression of genes involved in microbicidal responses induced by *P. aeruginosa*-secreted products were exclusively dependent on the *cftr* ΔF508 mutation. Indeed, the transfection of CF-TG cells with the *cftr* gene corrected their transcriptional profile toward a profile similar to that of wt TG cells. In a previous study, it has also been shown that the transfer of a functional *cftr* gene to CF-TG cells corrects defective secretory function [54]. These results clearly demonstrated that both inflammatory and non-microbicidal signatures of CF-TG cells were related to the expression of the *cftr* gene.

Finally, it has been recently demonstrated in *cftr*-deficient mice that sphingolipids are involved in CF pathogenesis through altered pH of intracellular vesicles [25]. We showed here that sphingolipid metabolism was altered in human CF-TG cells. Indeed, the analysis of microarray data showed that ceramidase and numerous related genes were down-modulated whereas sphingosine kinase and other related genes were up-regulated in CF-TG cells compared to wt TG cells. Ceramide is critical in sphingolipid metabolism. It is generated from sphingomyelin by the action of acid sphingomyelinase or *de novo* synthesis through the ceramide synthase. It may be transformed into sphingosine, ceramide-1-phosphate, glucosylceramide by different enzymes, and sphingomyelin in the Golgi apparatus. Ceramide may also be glycosylated in galactosyl-ceramide by a galactosyl-ceramide synthase that transfers galactose from a UDP-galactose donor [55]. We showed that galactosyl-ceramide accumulated in CF-TG cells compared to wt TG cells whereas the ceramide levels were similar in wt TG and CF-TG cells. Note that bacteria such as *Escherichia coli* [56] and *Helicobacter pylori* [57] selectively interact with galactosyl-ceramide, suggesting that increased levels of galactosyl-ceramide may favor the binding of *P. aeruginosa* to CF-TG cells. In *cftr*-deficient mice, ceramide accumulation is observed in the respiratory tract epithelium and submucosa [25]. The apparent discrepancies between our results and those of Teichgräber *et al.* [25] may be explained by the different methods of ceramide determination, the complex regulation of the ceramide pathway and the different cell types. Hence, alveolar epithelial cells of *cftr*-deficient mice do not accumulate ceramide [25]. Defective acidification of organelles in CF cell types may alter sphingolipid metabolism and, as a consequence, the expression of proinflammatory cytokines [25]. We reasoned that pH neutralization of vesicles may increase the expression of inflammatory genes in wt TG cells. For that purpose, we used bafilomycin A1, a specific inhibitor of V-ATPase involved in vesicle acidification and chloroquine, a weak base that diminishes CpG signaling by neutralizing endosomal pH [58]. The genes that encode the seven cytokines of the inflammatory signature of CF-TG cells were up-regulated in wt TG cells treated by alkalinizing agents compared to untreated wt TG cells. This result extended the observation that defective CFTR leads to defective acidification of intracellular organelles [25], [59]. It also demonstrated a functional link between pH rise and inflammatory cytokines independently of CF.

In conclusion, our study represents the first comprehensive analysis of gene expression in TG cells. It reveals an original program consisting of a constitutive and moderate inflammatory state of CF-TG cells likely due to altered sphingolipid metabolism. Defective microbicidal responses to *P. aeruginosa* of CF-TG cells were related, at least in part, to defective IFN-γ pathway. The inflammatory signature and the defective microbicidal signature of CF-TG cells were corrected when CF-TG cells were transfected with a functional *cftr* gene. They may be sufficient to account for host inefficiency in struggle against *P. aeruginosa* in cystic fibrosis. The candidate genes non-previously reported may serve as targets for new therapeutic approaches.

## Materials and Methods

### *P. aeruginosa*

The strain used in this study was isolated from a CF patient with a *cftr* ΔF508/ΔF508 genotype associated with an exocrine pancreatic insufficiency. It was the first isolated strain in sputum from this CF patient who was 16.2 years old at the time of collection. The strain belongs to the clone A lineage and has functional type IVa and flagellum tested by twitching and swimming mobility, respectively (data not shown). Single colonies isolated from CF and PAO1 reference [60] strains were grown in LB to a stationary phase at 37°C for 18 h. Bacterial suspensions (2×10^9^ cfu/ml) were centrifuged at 3,000×*g* for 15 min, and supernatant from the CF strain was further filtered through 0.2 µm filters and used to stimulate TG cells. Filtered LB was used as negative control.

### Analysis of *P. aeruginosa* products

*P. aeruginosa*-secreted products were submitted to tetraacetic acid precipitation for electrophoretic analysis and protein content determination. An equivalent of 0.025 OD_600_ units/µl was added to SDS-PAGE loading buffer. The samples were boiled for 10 min and the proteins separated by electrophoresis on a 12% polyacrylamide gel. The proteins were stained with Coomassie blue, cut out, subjected to procedures routinely performed in the proteomic platform at the IBSM (http://www.ibsm.cnrs-mrs.fr/ifrc/servtech/seq) and analyzed by mass spectrometry. Briefly, samples were treated via reduction and alkylation processes, digested with trypsin, desalted and subjected to mass spectrometry analysis using MALDI-TOF. Internal scale up was done with ions coming from trypsin autolysis and keratin. Mass values were compared with theoretical values from peptides referenced in databases.

### Cell culture

The serous tracheal gland cell lines MM39 (TG cells) and CF-KM4 (CF-TG cells) were isolated from a healthy donor [20] and a CF patient with a *cftr* ΔF508/ΔF508 mutation [19], respectively. Cell cultures were carried out as previously described [20], [61]. In brief, cells were maintained in Dulbecco's modified Eagle's/Ham's F12 medium (Sigma Chemicals) supplemented with 0.1% Ultroser G (Biosepra), 5% adult bovine serum, 5 g/l D-glucose and 0.1 g/l pyruvate. The absence of mycoplasma in cell cultures was checked using the VenorGeM kit (Biovalley). In some experiments, CF-TG cells were transfected with the pcDNA_3_-CFTR plasmid (Transgène, Strasbourg, France) or with the pM1-LUC plasmid (Roche Molecular Biology) as a negative control using the Lipofectamine Reagent (Invitrogen) as previously described [54]. Cells plated in 12-well tissue culture dishes (Falcon) were stimulated with *P. aeruginosa*-secreted products (1∶10 dilution) for 3 h for transcriptional studies, as described elsewhere [62], or 24 h for functional studies. In some experiments, 1000 UI of human recombinant IFNγ (Peprotech) were added to wt TG and CF-TG cells for 3 h before transcriptional analyses by real-time quantitative RT-PCR (qRT-PCR) (see below). The alkalinization of intracellular organelles was performed as previously described [58]. Wild type TG cells were incubated with different concentrations of bafilomycin A1 or chloroquine (Sigma) for 3 h, and the expression of inflammatory genes was studied by qRT-PCR.

### Microarrays and data analysis

Total RNA was extracted using the RNeasy minikit (Qiagen) and DNAse treatment. The quality and the quantity of RNA preparation were assessed using the 2100 Bioanalyzer and the RNA 6000 Nano LabChip kit (Agilent Technologies). The 4×44 k Human Whole Genome microarrays (Agilent Technologies) representing 33,581 genes or transcripts were used. Sample labeling and hybridization were performed according to protocols specified by the manufacturer (One-Color Microarray-Based Gene Expression Analysis). Briefly, 300 ng of total RNA and cyanine 3-labeled CTP (Cy-3) fluorescent dyes were used to generate fluorescent cRNA with Low RNA Input Fluorescent Amplification Kit (Agilent Technologies). Hybridization was performed for 17 h at 65°C using the *In situ* Hybridization Kit Plus (Agilent Technologies). All processing steps were performed in an ozone-controlled environment ([O3] <2 ppb). Twelve samples with three samples per cell group were included in the analysis. Slides were scanned at a 5 µm resolution with a G2505B DNA microarray scanner (Agilent Technologies). Image analysis and intra-array signal correction were performed using Agilent Feature Extractor Software A.9.1.3. Data processing, analysis and visualizing were performed using the Resolver software 7.1 (Rosetta Inpharmatics). Its error model-based transformation pipeline was used to map replicate reporters to genes and perform inter-array normalization. The intensity error model and its applications were used as described elsewhere [63]. The same procedure was used to discriminate the genes differentially expressed by two data groups (CF-TG cells *vs*. wt TG cells; wt TG stimulated cells *vs*. wt TG cells, CF-TG stimulated cells *vs*. CF-TG cells). Data were pre-processed by selecting only the best known genes (21,236) and then applying filters on gene expression level and variation among samples. Thus, a gene was kept for further analysis when the *P*-value of the error model was below 0.01 in at least 3 of the 6 samples and its coefficient of variation among samples was over 0.2. Discrimination between samples was performed using the unpaired Student's *t* test, the fold change (FC) and its associated *P*-value obtained with the Resolver error-model. A gene was considered as differentially expressed between two groups whether it met both criteria: a *P*-value for 2-sided Student's *t* test below 0.01 and an absolute |FC|>1.5 with a confidence *P*-value below 0.01. All data are MIAME compliant (accession no. A-MEXP-1332; www.ebi.ac.uk/miamexpress) [64]. Results were expressed as FC in fluorescence intensities between two groups, and expressed in log2. Gene families were determined using numerous databases: DAVID Bioinformatics Resources 2008 (http://david.abcc.ncifcrf.gov/), Online Medelian Inheritance in Man (http://www.ncbi.nlm.nih.gov/sites/entrez?db=OMIM&TabCmd=Limits), SOURCE (http://smd.stanford.edu/cgi-bin/source/sourceSearch), and Babelomics Fatigo+ (http://babelomics2.bioinfo.cipf.es/fatigoplus/cgi-bin/fatigoplus.cgi). The genes involved in the sphingolipid metabolism were studied using a construction that connected the 25 entities belonging to the sphingolipid-metabolism-process Gene Ontology group. Regulatory (dashed grey arrow) and physical (plain purple line) interactions between entities was based on information extracted from the literature using PathwayStudio™ (AriadneGenomics). Data from microarray experiments were collected and red color corresponded to up-regulated genes in CF-TG cells compared to wt TG cells and blue color to down-regulated genes.

### qRT-PCR

Reverse transcription of total RNA was performed according to the manufacturer's protocol (Invitrogen). Forward and reverse primers were constructed according to published sequences, and design was optimized for the LightCycler 480 System (Roche Diagnostics) (see Table S7 for primer sequences). qRT-PCR was performed using the LightCycler 480 SYBR Green I Master kit. Temperature cycling proceeded as follows: 5 min at 95°C to activate the FastStart Taq DNA Polymerase, followed by 45 cycles each consisting of: 15 s at 95°C for denaturation, followed by 30 s at 60°C for primer annealing and 30 s at 72°C for elongation. Melting curve analyses were performed by increasing the temperature from 70°C to 95°C. A standard curve for each primer was performed to determine the amplification efficiency. Relative quantification was calculated from the Cp values of the targeted gene and the reference gene using the LightCycler 480 software analysis (Roche Diagnostics). The mean ratio was calculated from triplicate assays. Each experiment was performed three times. Results were expressed as FC between two groups.

### Flow cytometry

Cells (5×10^5^ cells in 100 µl of phosphate buffered saline (PBS) containing 10% foetal calf serum and 1% sodium azide) were incubated with 10 µl of FITC-conjugated TLR2 (eBioscience) antibodies (Abs) for 1 h at 4°C or with 10 µl of IFNγR2 Abs (Santa Cruz) for 1 h followed by an incubation with 5 µl of swine anti-human IgG-FITC Abs (R&D Systems) for 30 min at 4°C. After washing, cells were analyzed by flow cytometry (EPICS XL, Beckman Coulter). Fluorescence was determined on all cells for each sample after debris, dead cells, and aggregates were excluded by selective gating based on orthogonal and side light scatter characteristics. Mean fluorescence intensity (MFI) was compared with control staining using an irrelevant isotype-matched human monoclonal antibody. For each sample, at least ten thousand events were collected and histograms were generated per sample. The percentage of positive cells was determined using the Expo32 ADC and the WinMDI 2.8 software. Each experiment was performed three times and a representative histogram is shown.

### ELISA

Cells (2×10^6^ cells) were stimulated with *P. aeruginosa*-secreted products for 24 h, centrifuged and supernatants were stored at −80°C before assays. The enzyme-linked immunobsorbent assays (ELISA) were provided from Beckman Coulter for IL-1α, R&D Systems for IL-1β, IL-24 and TNFSF14, RayBiotech for LIF and CXCL10, Preprotech for CXCL1, and Hycult Biotechnology for S100A8/S100A9. The assays were performed according to the manufacturer's instructions. The detection limit of the kits was less than 1.6 ng/ml for S100A8/S100A9, 15 pg/ml for IL-1α, LIF and IL-24, 10 pg/ml for CXCL1, 8 pg/ml for CXCL10, 5.5 pg/ml for TNFSF14, and 0.023 pg/ml for IL-1β. The mean ratio was calculated from triplicate assays. Each experiment was performed three times.

### Sphingolipid metabolism

Pelleted cells (2×10^7^ cells per assay) were suspended in 1 ml of water, and lipids were extracted by 20 ml of chloroform/methanol (2:1 volume), as already described [65]. After evaporation of the lipid phase under nitrogen, lipids were separated by four-stage, one-dimensional thin-layer chromatography (TLC) using 20×20 cm TLC Silica Gel G60 analytical plates (Merck), according to Augé et al. [66]. For staining, plates were dipped in 10% copper sulfate/8% phosphoric acid solution [64]. The plates were drained and heated at 130°C for 1 h. Several standard calibration curves were constructed with 0.5–50 µg of pure sphingolipid species (sphingomyelin, ceramides, galactocerebrosides, glucocerebrosides, lactocerebrosides, 1-β-D galactosylsphingosine) all species provided from Sigma. Ceramides and galactosyl-ceramides were quantified by densitometry after scanning the plates and using the software package Bio-1D^++^ (Vilber Lourmat).

### Statistical analysis

Results are expressed as means±SEM and [95% CI] and compared with the non-parametric Mann-Whitney *U* test. Statistical significance of qRT-PCR data was determined using the Student's *t* test. Differences were considered significant when *P*<0.05.

## Supporting Information

Table S1 Functional classification of up-regulated genes in CF-TG cells
(0.11 MB DOC)

Table S2 Functional classification of down-regulated genes in CF-TG cells
(0.12 MB DOC)

Table S3 Functional classification of up-regulated genes in P. aeruginosa-stimulated TG cells
(0.12 MB DOC)

Table S4 Functional classification of down-regulated genes in P. aeruginosa-stimulated TG cells
(0.07 MB DOC)

Table S5 Functional classification of up-regulated genes in P. aeruginosa-stimulated CF-TG cells
(0.11 MB DOC)

Table S6 Functional classification of down-regulated genes in P. aeruginosa-stimulated CF-TG cells
(0.07 MB DOC)

Table S7 Primers used for qRT-PCR
(0.04 MB DOC)
