# Supplementary material for: New Microbicidal Functions of Tracheal Glands: Defective Anti-Infectious Response to Pseudomonas aeruginosa in Cystic Fibrosis
Source: PLoS One. 2009 Apr 28;4(4):e5357. doi: 10.1371/journal.pone.0005357 (PMC2670521; doi:10.1371/journal.pone.0005357)
Supplement: Table S1 — Functional classification of up-regulated genes in CF-TG cells (0.11 MB DOC) [file pone.0005357.s001.doc]

**Table S1.** Functional classification of up-regulated genes in CF-TG cells

| **Category** | **Gene name** | | | **Symbol** | | **Fold Change** | **Accession No.** |
| --- | --- | --- | --- | --- | --- | --- | --- |
|  |  | | |  | |  |  |
| **Chemokines/ Cytokines/ Growth factors** | | | |  | |  |  |
| Interleukin 1. beta | | |  | | IL1B | 4.34 | NM_000576 |
| Interleukin 1. alpha | | |  | | IL1A | 3.80 | NM_000575 |
| Insulin-like 4 (placenta) | | |  | | INSL4 | 3.76 | NM_002195 |
| Chemokine (C-X-C motif) ligand 1 (melanoma growth stimulating activity. alpha) | | |  | | CXCL1 | 3.20 | NM_001511 |
| Interleukin 32. transcript variant 1 | | |  | | IL32 | 2.51 | NM_001012631 |
| Tumor necrosis factor (ligand) sup erfamily. member 14. transcript variant 1 | | |  | | TNFSF14 | 2.24 | NM_003807 |
| Chemokine (C-X-C motif) ligand 2 | | |  | | CXCL2 | 1.99 | NM_002089 |
| Leukemia inhibitory factor (cholinergic differentiation factor) | | |  | | LIF | 1.93 | NM_002309 |
| Fibroblast growth factor 2 (basic) | | |  | | FGF2 | 1.66 | NM_002006 |
|  |  | | |  | |  |  |
| **Inflammatory response** | | | |  | |  |  |
| Serine peptidase inhibitor. Kazal type 6 | | |  | | SPINK6 | 8.42 | NM_205841 |
| Aquaporin 9 | | |  | | AQP9 | 2.74 | NM_020980 |
| Mucin 20. cell surface associated | | |  | | MUC20 | 2.18 | NM_152673 |
| Thioredoxin interacting protein | | |  | | TXNIP | 2.02 | NM_006472 |
|  |  | | |  | |  |  |
| **Matrix remodeling** | | | |  | |  |  |
| Plasminogen activator. urokinase | | |  | | PLAU | 3.95 | NM_002658 |
| Serpin peptidase inhibitor. clade B (ovalbumin). member 4 | | |  | | SERPINB4 | 3.83 | NM_002974 |
| Serpin peptidase inhibitor. clade B (ovalbumin). member 3 | | |  | | SERPINB3 | 3.59 | NM_006919 |
| ADAM metallopeptidase domain 8 | | |  | | ADAM8 | 3.42 | NM_001109 |
| Chitinase 3-like 2. transcript variant 1 | | |  | | CHI3L2 | 3.40 | NM_004000 |
| Plasminogen activator. tissue. transcript variant 1 | | |  | | PLAT | 3.36 | NM_000930 |
| Carboxypeptidase Z. transcript variant 3 | | |  | | CPZ | 2.61 | NM_001014448 |
| HtrA serine peptidase 1 | | |  | | HTRA1 | 2.60 | NM_002775 |
| Heparan sulfate (glucosamine) 3-O-sulfotransferase 1 | | |  | | HS3ST1 | 2.39 | NM_005114 |
| Proteoglycan 1. secretory granule | | |  | | PRG1 | 2.04 | NM_002727 |
|  |  | | |  | |  |  |
| **Receptors/ Signal transduction** | | | |  | |  |  |
| SLIT and NTRK-like family. member 6 | | |  | | SLITRK6 | 5.52 | NM_032229 |
| Prostaglandin-endoperoxide synthase 2 (prostaglandin G/H synthase and cyclooxygenase) | | |  | | PTGS2 | 3.32 | NM_000963 |
| Roundabout homolog 4. magic roundabout (Drosophila) | | |  | | ROBO4 | 3.20 | NM_019055 |
| Cytochrome P450. family 27. subfamily B. polypeptide 1. nuclear gene encoding mitochondrial protein | | |  | | CYP27B1 | 2.58 | NM_000785 |
| Hematopoietic SH2 domain containing | | |  | | HSH2D | 2.59 | NM_032855 |
| Interferon regulatory factor 1 | | |  | | IRF1 | 2.40 | NM_002198 |
| Arachidonate 5-lipoxygenase | | |  | | ALOX5 | 2.35 | NM_000698 |
| SH2 domain containing 1B | | |  | | SH2D1B | 2.13 | NM_053282 |
| Guanine nucleotide binding protein (G protein). alpha 15 (Gq class) | | |  | | GNA15 | 2.09 | NM_002068 |
| Suppressor of cytokine signaling 3 | | |  | | SOCS3 | 2.06 | NM_003955 |
| Platelet-activating factor receptor | | |  | | PTAFR | 2.00 | NM_000952 |
| Receptor (calcitonin) activity modifying protein 1 | | |  | | RAMP1 | 1.87 | NM_005855 |
| Guanine nucleotide binding protein (G protein). gamma 11 | | |  | | GNG11 | 1.69 | NM_004126 |
|  |  | | |  | |  |  |
| **Transcription regulation** | | | |  | |  |  |
| Tripartite motif-containing 15. transcript variant 1 | | |  | | TRIM15 | 3.53 | NM_033229 |
| Basic leucine zipper transcription factor. ATF-like | | |  | | BATF | 2.90 | NM_006399 |
| Zinc finger protein 462 | | |  | | ZNF462 | 2.22 | NM_021224 |
| Snail homolog 1 (Drosophila) | | |  | | SNAI1 | 2.08 | NM_005985 |
| 2'-5'-oligoadenylate synthetase-like. transcript variant 1 | | |  | | OASL | 1.97 | NM_003733 |
| Runt-related transcription factor 3. transcript variant 2 | | |  | | RUNX3 | 1.88 | NM_004350 |
| Nuclear factor of kappa light polypeptide gene enhancer in B-cells inhibitor. epsilon | | |  | | NFKBIE | 1.86 | NM_004556 |
| Nuclear receptor subfamily 1. group I. member 3 | | |  | | NR1I3 | 1.86 | NM_001077482 |
| Estrogen-related receptor alpha | | |  | | ESRRA | 1.80 | NM_004451 |
| GATA binding protein 3. transcript variant 1 | | |  | | GATA3 | 1.76 | NM_001002295 |
| PHD finger protein 15 | | |  | | PHF15 | 1.64 | AK025001 |
|  | | |  | |  |  |  |
| **Adhesion/ Cytoskeleton/ Cell communication** | | | |  | |  |  |
| Vimentin | | |  | | VIM | 2.65 | NM_003380 |
| Villin-like | | |  | | VILL | 2.50 | NM_015873 |
| Keratin. hair. acidic. 4 | | |  | | KRT34 | 2.35 | NM_021013 |
| Keratin. hair. basic. 3 | | |  | | KRT83 | 2.18 | NM_002282 |
| Intercellular adhesion molecule 1 (CD54). human rhinovirus receptor | | |  | | ICAM1 | 2.15 | NM_000201 |
| Neural precursor cell expressed. developmentally down-regulated 9. transcript variant 1 | | |  | | NEDD9 | 1.74 | NM_006403 |
| Myosin regulatory light chain interacting protein | | |  | | MYLIP | 1.71 | NM_013262 |
|  | | | |  | |  |  |
| **Transport/ Ion Transport** | | |  | |  |  |  |
| Potassium channel tetramerisation domain containing 14 | | |  | | KCTD14 | 2.79 | NM_023930 |
| Pleckstrin homology domain containing. family G (with RhoGef domain) member 3 | | |  | | PLEKHG3 | 2.72 | NM_015549 |
| Zinc finger. BED-type containing 2 | | |  | | ZBED2 | 2.21 | NM_024508 |
| SPARC related modular calcium binding 1. transcript variant 1 | | |  | | SMOC1 | 2.21 | NM_001034852 |
| WNK lysine deficient protein kinase 4 | | |  | | WNK4 | 2.00 | NM_032387 |
| Solute carrier family 37 (glycerol-3-phosphate transporter). member 1 | | |  | | SLC37A1 | 1.91 | NM_018964 |
|  | |  | |  | |  |  |
| **Cell cycle/ Proliferation** | | | |  | |  |  |
| Wingless-type MMTV integration site family. member 5B. transcript variant 2 | | |  | | WNT5B | 2.05 | NM_030775 |
| Discoidin domain receptor family. member 2. transcript variant 1 | | |  | | DDR2 | 2.02 | NM_001014796 |
| Chordin-like 2 | | |  | | CHRDL2 | 1.69 | NM_015424 |
|  |  | | |  | |  |  |
| **Metabolism** | | | |  | |  |  |
| Hydroxy-delta-5-steroid dehydrogenase. 3 beta- and steroid delta-isomerase 1 | | |  | | HSD3B1 | 7.81 | NM_000862 |
| Thyroid peroxidase. transcript variant 1 | | |  | | TPO | 3.00 | NM_000547 |
| ATP-binding cassette. sub-family G (WHITE). member 1. transcript variant 1 | | |  | | ABCG1 | 2.16 | NM_207630 |
| Threonyl-tRNA synthetase-like 2 | | |  | | TARSL2 | 1.98 | NM_152334 |
| Aspartate beta-hydroxylase domain containing 1 | | |  | | ASPHD1 | 1.92 | NM_181718 |
| guanine deaminase | | |  | | GDA | 1.70 | NM_004293 |
