# Supplementary material for: New Microbicidal Functions of Tracheal Glands: Defective Anti-Infectious Response to Pseudomonas aeruginosa in Cystic Fibrosis
Source: PLoS One. 2009 Apr 28;4(4):e5357. doi: 10.1371/journal.pone.0005357 (PMC2670521; doi:10.1371/journal.pone.0005357)
Supplement: Table S2 — Functional classification of down-regulated genes in CF-TG cells (0.12 MB DOC) [file pone.0005357.s002.doc]

**Table S2.** Functional classification of down-regulated genes in CF-TG cells

| **Category** | **Gene name** | | | **Symbol** | | **Fold Change** | **Accession No.** | |
| --- | --- | --- | --- | --- | --- | --- | --- | --- |
| **Chemokines/ Cytokines/ Growth factors** | | | |  | |  |  | |
| Platelet derived growth factor C | | |  | | PDGFC | -1.62 | NM_016205 |  |
|  | | |  | |  |  |  |  |
| **Inflammatory response** | | | |  | |  |  | |
| Complement factor I | | |  | | CFI | -4.09 | NM_000204 | |
|  |  | | |  | |  |  | |
| **Receptors/ Signal transduction** | | | |  | |  |  | |
| Guanine nucleotide binding protein (G protein), beta polypeptide 4 | | |  | | GNB4 | -3.93 | NM_021629 | |
| Sarcoglycan, epsilon | | |  | | SGCE | -3.73 | NM_003919 | |
| Fyn-related kinase | | |  | | FRK | -2.67 | NM_002031 | |
| G protein-coupled receptor 103 | | |  | | GPR103 | -2.63 | NM_198179 | |
| Phospholipase C, epsilon 1 | | |  | | PLCE1 | -2.35 | NM_016341 | |
| Protein tyrosine phosphatase-like A domain containing 2 | | |  | | PTPLAD2 | -2.27 | NM_001010915 | |
| Transmembrane protein 98, transcript variant 1 | | |  | | TMEM98 | -2.22 | NM_015544 | |
| G protein-coupled receptor 155, transcript variant 2 | | |  | | GPR155 | -2.20 | NM_152529 | |
| Mitogen-activated protein kinase kinase kinase 4 | | |  | | MAP3K4 | -2.09 | AK094629 | |
| Dual specificity phosphatase 12 | | |  | | DUSP12 | -1.99 | BM474343 | |
| Phospholipase A2, group IVA (cytosolic, calcium-dependent) | | |  | | PLA2G4A | -1.89 | NM_024420 | |
| Protein phosphatase 1, regulatory (inhibitor) subunit 3C | | |  | | PPP1R3C | -1.85 | NM_005398 | |
| Interleukin 6 receptor, transcript variant 1 | | |  | | IL6R | -1.74 | NM_000565 | |
| Phosphatidylinositol glycan, class Z | | |  | | PIGZ | -1.73 | NM_025163 | |
| GTP-binding protein 8 (putative), transcript variant 1 | | |  | | GTPBP8 | -1.67 | NM_014170 | |
| Receptor (TNFRSF)-interacting serine-threonine kinase 1 | | |  | | RIPK1 | -1.63 | NM_003804 | |
| Olfactory receptor, family 7, subfamily E, member 13 pseudogene | | |  | | OR7E13P | -1.62 | AF238487 | |
| Cytochrome P450, family 4, subfamily V, polypeptide 2 | | |  | | CYP4V2 | -1.58 | NM_207352 | |
| Copine VIII | | |  | | CPNE8 | -1.58 | NM_153634 | |
| Protein kinase C, alpha | | |  | | PRKCA | -1.56 | NM_002737 | |
| Transmembrane protein 107, transcript variant 1 | | |  | | TMEM107 | -1.56 | NM_032354 | |
| Adaptor-related protein complex 1, sigma 2 subunit | | |  | | AP1S2 | -1.52 | NM_003916 | |
|  |  | | |  | |  |  | |
| **Transcription regulation** | | | |  | |  |  | |
| Zinc finger, CCHC domain containing 2 | | |  | | ZCCHC2 | -2.09 | NM_017742 | |
| PC4 and SFRS1 interacting protein 1, transcript variant 1 | | |  | | PSIP1 | -1.86 | NM_021144 | |
| Forkhead box F2 | | |  | | FOXF2 | -1.84 | NM_001452 | |
| Regulator of chromosome condensation (RCC1) and BTB (POZ) domain containing protein 2 | | |  | | RCBTB2 | -1.73 | NM_001268 | |
| LIM homeobox 2 | | |  | | LHX2 | -1.71 | NM_004789 | |
| Jun D proto-oncogene | | |  | | JUND | -1.71 | X56681 | |
| Pirin (iron-binding nuclear protein), transcript variant 1 | | |  | | PIR | -1.71 | NM_003662 | |
| Sirtuin (silent mating type information regulation 2 homolog) 5 (S. cerevisiae), transcript variant 1 | | |  | | SIRT5 | -1.71 | NM_012241 | |
| Mediator of RNA polymerase II transcription, subunit 28 homolog (S. cerevisiae) | | |  | | MED28 | -1.69 | NM_025205 | |
| Zinc finger protein 701 | | |  | | ZNF701 | -1.69 | NM_018260 | |
| Basonuclin 2 | | |  | | BNC2 | -1.65 | NM_017637 | |
| Staufen, RNA binding protein, homolog 2 (Drosophila) | | |  | | STAU2 | -1.59 | NM_014393 | |
| PC4 and SFRS1 interacting protein 1, transcript variant 2 | | |  | | PSIP1 | -1.56 | NM_033222 | |
|  | | |  | |  |  |  | |
| **Adhesion/ Cytoskeleton/ Cell communication** | | | |  | |  |  | |
| WAS protein family, member 3 | | |  | | WASF3 | -3.89 | NM_006646 | |
| MAP/microtubule affinity-regulating kinase 1 | | |  | | MARK1 | -2.91 | NM_018650 | |
| CNKSR family member 3 | | |  | | CNKSR3 | -2.67 | NM_173515 | |
| sorting nexin 10 | | |  | | SNX10 | -2.58 | NM_013322 | |
| PREDICTED: family with sequence similarity 7, member A3, transcript variant 3 | | |  | | FAM7A3 | -2.55 | XM_930891 | |
| Activity-regulated cytoskeleton-associated protein | | |  | | ARC | -2.05 | NM_015193 | |
| Tubulin tyrosine ligase-like family, member 7 | | |  | | TTLL7 | -1.96 | NM_024686 | |
| Collagen, type XIV, alpha 1 (undulin) | | |  | | COL14A1 | -1.91 | M64109 | |
| BMP and activin membrane-bound inhibitor homolog (Xenopus laevis) | | |  | | BAMBI | -1.91 | NM_012342 | |
| Rho guanine nucleotide exchange factor (GEF) 15 | | |  | | ARHGEF15 | -1.90 | NM_173728 | |
| Synapse defective 1, Rho GTPase, homolog 2 (C. elegans) | | |  | | FLJ13815 | -1.86 | AK075186 | |
| HUS1 checkpoint homolog (S. pombe) | | |  | | HUS1 | -1.80 | NM_004507 | |
| Spectrin domain with coiled-coils 1, transcript variant NSP5beta3beta | | |  | | SPECC1 | -1.63 | NM_001033553 | |
| Internexin neuronal intermediate filament protein, alpha | | |  | | INA | -1.61 | NM_032727 | |
| Calcium/calmodulin-dependent protein kinase II inhibitor 1 | | |  | | CaMKIINalpha | -1.59 | BC020630 | |
| Family with sequence similarity 63, member A, transcript variant 1 | | |  | | FAM63A | -1.58 | NM_018379 | |
| AF262221 SNARE Vti1a protein (Rattus norvegicus) | | |  | | THC2310680 | -1.57 | THC2310680 | |
| Meiosis-specific nuclear structural 1 | | |  | | MNS1 | -1.57 | NM_018365 | |
|  |  | | |  | |  |  | |
| **Transport/ Ion Transport** | |  | |  | |  |  | |
| Chaperonin containing TCP1, subunit 6B (zeta 2) | | |  | | CCT6B | -2.20 | NM_006584 | |
| Calcyphosine 2 | | |  | | CAPS2 | -2.12 | AK091839 | |
| Ring finger protein 130 | | |  | | RNF130 | -1.82 | NM_018434 | |
| Heme binding protein 1 | | |  | | HEBP1 | -1.74 | NM_015987 | |
| Solute carrier family 30 (zinc transporter), member 1 | | |  | | SLC30A1 | -1.74 | NM_021194 | |
| Leucine rich repeat containing 28 | | |  | | LRRC28 | -1.62 | NM_144598 | |
| Solute carrier family 35, member F5 | | |  | | SLC35F5 | -1.53 | AK025657 | |
|  | | | |  | |  |  | |
| **Cell cycle/ Proliferation** | | | |  | |  |  | |
| Sno, strawberry notch homolog 1 (Drosophila) | | |  | | SBNO1 | -1.70 | AK074256 | |
| H-rev107-like protein 5 | | |  | | HRLP5 | -1.70 | BC034222 | |
| HIRA interacting protein 5, transcript variant 1 | | |  | | HIRIP5 | -1.52 | NM_015700 | |
|  |  | | |  | |  |  | |
| **Metabolism** | | | |  | |  |  | |
| Methionine sulfoxide reductase B3, transcript variant 1 | | |  | | MSRB3 | -6.65 | NM_198080 | |
| Heme oxygenase (decycling) 1 | | |  | | HMOX1 | -2.39 | NM_002133 | |
| Galactosylceramidase, transcript variant 1 | | |  | | GALC | -2.27 | NM_000153 | |
| Methylenetetrahydrofolate dehydrogenase (NADP+ dependent) 1-like | | |  | | MTHFD1L | -2.12 | AY374131 | |
| N-acetylneuraminate pyruvate lyase (dihydrodipicolinate synthase) | | |  | | NPL | -2.10 | NM_030769 | |
| Acyl-Coenzyme A dehydrogenase, short/branched chain, nuclear gene encoding mitochondrial protein | | |  | | ACADSB | -1.91 | NM_001609 | |
| Histone 1, H4k | | |  | | HIST1H4K | -1.90 | NM_003541 | |
| Aminoadipate-semialdehyde synthase | | |  | | AASS | -1.82 | NM_005763 | |
| Hedgehog acyltransferase | | |  | | HHAT | -1.73 | NM_018194 | |
| Glucosaminyl (N-acetyl) transferase 1, core 2 (beta-1,6-N-acetylglucosaminyltransferase) | | |  | | GCNT1 | -1.72 | NM_001490 | |
| Establishment of cohesion 1 homolog 2 (S. cerevisiae) | | |  | | ESCO2 | -1.72 | NM_001017420 | |
| Malic enzyme 1, NADP(+)-dependent, cytosolic | | |  | | ME1 | -1.67 | NM_002395 | |
| Histidyl-tRNA synthetase 2 | | |  | | HARS2 | -1.66 | NM_080820 | |
| Thiamin pyrophosphokinase 1, transcript variant 1 | | |  | | TPK1 | -1.64 | NM_022445 | |
| Similar to CG11994-PA | | |  | | ADAL | -1.62 | BX647599 | |
| Oxysterol binding protein-like 1A, transcript variant OSBPL1B | | |  | | OSBPL1A | -1.62 | NM_080597 | |
| SEC14-like 1 (S. cerevisiae) | | |  | | SEC14L1 | -1.55 | AF130050 | |
| Glutathione S-transferase A4 | | |  | | GSTA4 | -1.55 | NM_001512 | |
| Pyroglutamyl-peptidase I | | |  | | PGPEP1 | -1.54 | NM_017712 | |
| Polymerase (DNA directed), alpha | | |  | | POLA | -1.52 | NM_016937 | |
|  |  | | |  | |  |  | |
| **Protein degradation** | | | |  | |  |  | |
| E3 ubiquitin protein ligase, HECT domain containing, 1 | | |  | | EDD | -2.94 | AK095151 | |
| F-box protein 32, transcript variant 1 | | |  | | FBXO32 | -2.48 | NM_058229 | |
| F-box and leucine-rich repeat protein 17 | | |  | | FBXL17 | -1.97 | BC018548 | |
