# Supplementary material for: New Microbicidal Functions of Tracheal Glands: Defective Anti-Infectious Response to Pseudomonas aeruginosa in Cystic Fibrosis
Source: PLoS One. 2009 Apr 28;4(4):e5357. doi: 10.1371/journal.pone.0005357 (PMC2670521; doi:10.1371/journal.pone.0005357)
Supplement: Table S3 — Functional classification of up-regulated genes in P. aeruginosa-stimulated TG cells (0.12 MB DOC) [file pone.0005357.s003.doc]

**Table S3.** Functional classification of up-regulated genes in *P. aeruginosa*-stimulated TG cells

| **Category** | **Gene name** | | | **Symbol** | | **Fold Change** | **Accession No.** |
| --- | --- | --- | --- | --- | --- | --- | --- |
| **Chemokines/ Cytokines/ Growth factors** | | | |  | |  |  |
| Chemokine (C-X-C motif) ligand 10 | | |  | | CXCL10 | 49.82 | NM_001565 |
| Chemokine (C-X-C motif) ligand 1 (melanoma growth stimulating activity, alpha) | | |  | | CXCL1 | 18.65 | NM_001511 |
| Interleukin 1, beta | | |  | | IL1B | 9.04 | NM_000576 |
| Interleukin 1, alpha | | |  | | IL1A | 6.26 | NM_000575 |
| Leukemia inhibitory factor (cholinergic differentiation factor) | | |  | | LIF | 6.17 | NM_002309 |
| Interleukin 1 family, member 9 | | |  | | IL1F9 | 6.02 | NM_019618 |
| Interleukin 32 | | |  | | IL32 | 5.07 | NM_001012631 |
| Insulin-like growth factor 2 (somatomedin A), transcript variant 2 | | |  | | IGF2 | 5.00 | NM_001007139 |
| Colony stimulating factor 2 (granulocyte-macrophage) | | |  | | CSF2 | 4.43 | NM_000758 |
| Interferon, kappa | | |  | | IFNK | 3.91 | NM_020124 |
| Chemokine (C-C motif) ligand 20 | | |  | | CCL20 | 3.74 | NM_004591 |
| Chemokine (C-X-C motif) ligand 11 | | |  | | CXCL11 | 3.61 | NM_005409 |
| Tumor necrosis factor (TNF superfamily, member 2) | | |  | | TNF | 3.48 | NM_000594 |
| Tumor necrosis factor (ligand) superfamily, member 14, transcript variant 1 | | |  | | TNFSF14 | 2.82 | NM_003807 |
| Transforming growth factor, alpha | | |  | | TGFA | 2.68 | NM_003236 |
| Colony stimulating factor 3 (granulocyte), transcript variant 1 | | |  | | CSF3 | 2.47 | NM_000759 |
| Interleukin 24, transcript variant 1 | | |  | | IL24 | 2.34 | NM_006850 |
|  |  | | |  | |  |  |
| **Inflammatory response** | | | |  | |  |  |
| Pentraxin-related gene, rapidly induced by IL-1 beta | | |  | | PTX3 | 7.07 | NM_002852 |
| Superoxide dismutase 2, mitochondrial, nuclear gene encoding mitochondrial protein, transcript variant 1 | | |  | | SOD2 | 4.92 | NM_000636 |
| Complement factor B | | |  | | CFB | 4.29 | NM_001710 |
| S100 calcium binding protein A8 (calgranulin A) | | |  | | S100A8 | 3.94 | NM_002964 |
| Defensin, beta 4 | | |  | | DEFB4 | 3.88 | NM_004942 |
| Peptidase inhibitor 3, skin-derived | | |  | | PI3 | 3.64 | NM_002638 |
| S100 calcium binding protein A12 (calgranulin C) | | |  | | S100A12 | 3.33 | NM_005621 |
| S100 calcium binding protein A7 (psoriasin 1) | | |  | | S100A7 | 3.17 | NM_002963 |
| Defensin, beta 103A | | |  | | DEFB103A | 3.15 | NM_018661 |
| S100 calcium binding protein A9 (calgranulin B) | | |  | | S100A9 | 2.98 | NM_002965 |
| Thrombomodulin | | |  | | THBD | 2.74 | NM_000361 |
| S100 calcium binding protein A3 | | |  | | S100A3 | 2.63 | NM_002960 |
|  |  | | |  | |  |  |
| **Matrix remodeling** | | | |  | |  |  |
| Plasminogen activator, urokinase | | |  | | PLAU | 7.98 | NM_002658 |
| Serpin peptidase inhibitor, clade B (ovalbumin), member 3 | | |  | | SERPINB3 | 5.19 | NM_006919 |
| Serpin peptidase inhibitor, clade B (ovalbumin), member 4 | | |  | | SERPINB4 | 4.93 | NM_002974 |
| Matrix metallopeptidase 1 (interstitial collagenase) | | |  | | MMP1 | 4.11 | NM_002421 |
| Matrix metallopeptidase 3 (stromelysin 1, progelatinase) | | |  | | MMP3 | 3.47 | NM_002422 |
|  |  | | |  | |  |  |
| **Receptor/ Signal transduction** | | | |  | |  |  |
| Cytochrome P450, family 1, subfamily A, polypeptide 1 | |  | | CYP1A1 | | 7.58 | NM_000499 |
| Tumor necrosis factor, alpha-induced protein 2 | |  | | TNFAIP2 | | 6.21 | NM_006291 |
| Oxidised low density lipoprotein (lectin-like) receptor 1 | |  | | OLR1 | | 5.27 | NM_002543 |
| Mitogen-activated protein kinase kinase kinase 8 | |  | | MAP3K8 | | 4.34 | NM_005204 |
| Transmembrane protein 46 | |  | | TMEM46 | | 4.23 | NM_001007538 |
| Adrenergic, beta-2-, receptor, surface | |  | | ADRB2 | | 3.41 | NM_000024 |
| CD83 molecule | |  | | CD83 | | 3.36 | NM_004233 |
| Major facilitator superfamily domain containing 2 | |  | | MFSD2 | | 3.25 | NM_032793 |
| Interferon gamma receptor 2 (interferon gamma transducer 1) | |  | | IFNGR2 | | 3.23 | NM_005534 |
| G protein-coupled receptor 110, transcript variant 2 | |  | | GPR110 | | 3.17 | NM_025048 |
| TNFAIP3 interacting protein 1 | |  | | TNIP1 | | 2.92 | NM_006058 |
| Suppressor of cytokine signaling 3 | |  | | SOCS3 | | 2.72 | NM_003955 |
| Cytochrome P450, family 27, subfamily B, polypeptide 1, nuclear gene encoding mitochondrial protein | |  | | CYP27B1 | | 2.66 | NM_000785 |
| Mitogen-activated protein kinase kinase 3, transcript variant C | |  | | MAP2K3 | | 2.65 | NM_145110 |
| Toll-like receptor 2 | |  | | TLR2 | | 2.63 | NM_003264 |
| Syndecan 4 (amphiglycan, ryudocan) | |  | | SDC4 | | 2.60 | NM_002999 |
| Phosphatidylinositol glycan anchor biosynthesis, class A (paroxysmal nocturnal hemoglobinuria), transcript variant 1 | |  | | PIGA | | 2.30 | NM_002641 |
|  |  | | |  | |  |  |
| **Transcription regulation** | | | |  | |  |  |
| Histone deacetylase 9, transcript variant 3 | |  | | HDAC9 | | 8.91 | NM_014707 |
| Apolipoprotein B mRNA editing enzyme, catalytic polypeptide-like 3A | |  | | APOBEC3A | | 6.25 | NM_145699 |
| Ets homologous factor | |  | | EHF | | 6.16 | NM_012153 |
| Zinc finger CCCH-type containing 12A | |  | | ZC3H12A | | 5.37 | NM_025079 |
| Nuclear factor of kappa light polypeptide gene enhancer in B-cells 1 (p105) | |  | | NFKB1 | | 4.29 | NM_003998 |
| Tripartite motif-containing 15, transcript variant 1 | |  | | TRIM15 | | 3.40 | NM_033229 |
| Testis nuclear RNA-binding protein-like | |  | | LOC161931 | | 3.11 | NM_139174 |
| Pre-B-cell colony enhancing factor 1, transcript variant 2 | |  | | PBEF1 | | 2.82 | NM_182790 |
| Tripartite motif-containing 36, transcript variant 1 | |  | | TRIM36 | | 2.80 | NM_018700 |
| Disrupted in schizophrenia 1, transcript variant L | |  | | DISC1 | | 2.34 | NM_018662 |
|  |  | | |  | |  |  |
| **Adhesion/ Cytoskeleton/ Cell communication** | | | |  | |  |  |
| Keratin 6B | |  | | KRT6B | | 6.42 | NM_005555 |
| Laminin, alpha 2 (merosin, congenital muscular dystrophy) | |  | | LAMA2 | | 3.22 | NM_000426 |
| Integrin, beta 8 | |  | | ITGB8 | | 2.94 | NM_002214 |
| Ninjurin 1 | |  | | NINJ1 | | 2.93 | NM_004148 |
|  |  | | |  | |  |  |
| **Transport/ Ion Transport** | | | |  | |  |  |
| Solute carrier family 28 (sodium-coupled nucleoside transporter), member 3 | |  | | SLC28A3 | | 6.24 | NM_022127 |
| TCDD-inducible poly(ADP-ribose) polymerase | |  | | TIPARP | | 4.12 | NM_015508 |
| Zinc finger, BED-type containing 2 | |  | | ZBED2 | | 3.18 | NM_024508 |
| Solute carrier organic anion transporter family, member 3A1 | |  | | SLCO3A1 | | 2.80 | NM_013272 |
| CUB and zona pellucida-like domains 1 | |  | | CUZD1 | | 2.10 | NM_022034 |
|  |  | | |  | |  |  |
| **Cell cycle/ Proliferation** | | | |  | |  |  |
| START domain containing 13 | |  | | STARD13 | | 3.22 | NM_178006 |
|  | |  | |  | |  |  |
| **Apoptosis** | |  | |  | |  |  |
| Caspase recruitment domain family, member 15 | |  | | CARD15 | | 2.78 | NM_022162 |
|  | |  | |  | |  |  |
| **Metabolism** | | | |  | |  |  |
| Vanin 3, transcript variant 1 | |  | | VNN3 | | 4.91 | NM_018399 |
| Vanin 1 | |  | | VNN1 | | 3.92 | NM_004666 |
| Protease, serine, 22 | |  | | PRSS22 | | 2.99 | NM_022119 |
| ELOVL family member 7, elongation of long chain fatty acids (yeast) | |  | | ELOVL7 | | 2.96 | NM_024930 |
| GTP cyclohydrolase 1 (dopa-responsive dystonia), transcript variant 1 | |  | | GCH1 | | 2.92 | NM_000161 |
| Acyl-CoA synthetase long-chain family member 5, transcript variant 3 | |  | | ACSL5 | | 2.41 | NM_203380 |
