# Supplementary material for: New Microbicidal Functions of Tracheal Glands: Defective Anti-Infectious Response to Pseudomonas aeruginosa in Cystic Fibrosis
Source: PLoS One. 2009 Apr 28;4(4):e5357. doi: 10.1371/journal.pone.0005357 (PMC2670521; doi:10.1371/journal.pone.0005357)
Supplement: Table S4 — Functional classification of down-regulated genes in P. aeruginosa-stimulated TG cells (0.07 MB DOC) [file pone.0005357.s004.doc]

**Table S4.** Functional classification of down-regulated genes in *P. aeruginosa*-stimulated TG cells

| **Category** | **Gene name** | | | **Symbol** | **Fold Change** | **Accession No.** |
| --- | --- | --- | --- | --- | --- | --- |
| **Receptors/Signal transduction** | | | |  |  |  |
| Mitogen-activated protein kinase 4 | | |  | MAPK4 | -2.01 | X59727 |
| Protein phosphatase 1. regulatory (inhibitor) subunit 3C | | |  | PPP1R3C | -1.97 | NM_005398 |
| Pleckstrin homology domain containing. family A member 6 | | |  | PLEKHA6 | -1.56 | NM_014935 |
| Olfactory receptor. family 7. subfamily E. member 13 pseudogene | | |  | OR7E13P | -1.07 | AF238487 |
|  | |  | |  |  |  |
| **Transcription regulation** | | | |  |  |  |
| 2'-5'-oligoadenylate synthetase-like. transcript variant 1 | | |  | OASL | -2.77 | NM_003733 |
| Zinc finger protein 70 | | |  | ZNF70 | -1.87 | NM_021916 |
| Seryl-tRNA synthetase | | |  | SARS | -1.75 | AK022339 |
| Similar to CG11994-PA | | |  | ADAL | -1.71 | BX647599 |
| Zinc finger protein 488 | | |  | ZNF488 | -1.71 | NM_153034 |
| RNA binding motif protein 35B | | |  | RBM35B | -1.53 | NM_024939 |
| Microphthalmia-associated transcription factor | | |  | MITF | -1.40 | AA778291 |
| PHD finger protein 6. transcript variant 3 | | |  | PHF6 | -1.21 | NM_032335 |
| Adenosine deaminase-like | | |  | ADAL | -1.20 | NM_001012969 |
| Kelch repeat and BTB (POZ) domain containing 11 | | |  | KBTBD11 | -1.14 | NM_014867 |
| Peroxisome proliferative activated receptor. alpha | | |  | PPARA | -1.13 | L02932 |
| Homeobox containing 1 | | |  | HMBOX1 | -1.07 | NM_024567 |
|  | |  | |  |  |  |
| **Cytoskeleton/ Cell communication** | | | |  |  |  |
| Ankyrin repeat domain 38 | | |  | ANKRD38 | -3.02 | NM_181712 |
| LINE-1 type transposase domain containing 1 | | |  | L1TD1 | -2.02 | NM_019079 |
| Gap junction protein. alpha 5. 40kDa (connexin 40). transcript variant A | | |  | GJA5 | -1.83 | NM_005266 |
| Coronin. actin binding protein. 2A. transcript variant 1 | | |  | CORO2A | -1.50 | NM_003389 |
| Lin-7 homolog B (C. elegans) | | |  | LIN7B | -1.21 | NM_022165 |
| Paxillin | | |  | PXN | -1.10 | CR626729 |
|  | |  | |  |  |  |
| **Transport** | | | |  |  |  |
| PQ loop repeat containing 2 | | |  | PQLC2 | -1.79 | AK000327 |
| HSPC049 protein | | |  | HSPC049 | -1.58 | NM_014149 |
| Solute carrier family 2 (facilitated glucose transporter). member 14 | | |  | SLC2A14 | 1.06 | BC060766 |
|  | |  | |  |  |  |
| **Cell cycle/ Proliferation** | | | |  |  |  |
| BRCA1 interacting protein C-terminal helicase 1 | | |  | BRIP1 | -2.94 | AK074713 |
| Nudix (nucleoside diphosphate linked moiety X)-type motif 16 | | |  | NUDT16 | -1.57 | NM_152395 |
| HECT domain containing 3 | | |  | HECTD3 | -1.06 | NM_024602 |
|  | |  | |  |  |  |
| **Apoptosis** | | | |  |  |  |
| Caspase 9. apoptosis-related cysteine peptidase. transcript variant alpha | |  | | CASP9 | -1.99 | NM_001229 |
|  | |  | |  |  |  |
| **Metabolism** | | | |  |  |  |
| Seryl-tRNA synthetase | |  | | SARS | -1.63 | AK022339 |
| Serine palmitoyltransferase. long chain base subunit 2-like (aminotransferase 2) | |  | | SPTLC2L | -1.58 | AK075271 |
|  | |  | |  |  |  |
| **Protein degradation** | | | |  |  |  |
| Ubiquitin specific peptidase 30 | |  | | USP30 | -1.87 | AL834278 |
| HECT domain containing 3 | |  | | HECTD3 | -1.62 | NM_024602 |
