# Supplementary material for: New Microbicidal Functions of Tracheal Glands: Defective Anti-Infectious Response to Pseudomonas aeruginosa in Cystic Fibrosis
Source: PLoS One. 2009 Apr 28;4(4):e5357. doi: 10.1371/journal.pone.0005357 (PMC2670521; doi:10.1371/journal.pone.0005357)
Supplement: Table S5 — Functional classification of up-regulated genes in P. aeruginosa-stimulated CF-TG cells (0.11 MB DOC) [file pone.0005357.s005.doc]

**Table S5.** Functional classification of up-regulated genes in *P. aeruginosa*-stimulated CF-TG cells

| **Category** | **Gene name** | | | **Symbol** | | | **Fold Change** | **Accession No.** |
| --- | --- | --- | --- | --- | --- | --- | --- | --- |
| **Chemokines/ Cytokines/ Growth factors** | | | |  | | |  |  |
| Chemokine (C-X-C motif) ligand 1 (melanoma growth stimulating activity, alpha) | |  | | CXCL1 | | | 7.56 | NM_001511 |
| Interleukin 1, beta | |  | | IL1B | | | 6.06 | NM_000576 |
| Interleukin 1, alpha | |  | | IL1A | | | 4.27 | NM_000575 |
| Leukemia inhibitory factor (cholinergic differentiation factor) | |  | | LIF | | | 4.13 | NM_002309 |
| Tumor necrosis factor (TNF superfamily, member 2) | |  | | TNF | | | 3.93 | NM_000594 |
| Interleukin 32, transcript variant 1 | |  | | IL32 | | | 3.06 | NM_001012631 |
| interleukin 8 | |  | | IL8 | | | 2.95 | NM_000584 |
| Colony stimulating factor 3 (granulocyte), transcript variant 1 | |  | | CSF3 | | | 2.86 | NM_000759 |
| Chemokine (C-C motif) ligand 20 | |  | | CCL20 | | | 2.74 | NM_004591 |
| Chemokine (C-X-C motif) ligand 2 | |  | | CXCL2 | | | 2.50 | NM_002089 |
| Tumor necrosis factor (ligand) superfamily, member 14, transcript variant 1 | |  | | TNFSF14 | | | 2.42 | NM_003807 |
| Interleukin 1 receptor antagonist, transcript variant 1 | |  | | IL1RN | | | 2.02 | NM_173842 |
|  |  | | |  | | |  |  |
| **Inflammatory response** | | | |  | | |  |  |
| Superoxide dismutase 2, mitochondrial, nuclear gene encoding mitochondrial protein, transcript variant 1 | | | | SOD2 | | | 3.33 | NM_000636 |
| Lipocalin 2 (oncogene 24p3) | | |  | | LCN2 | | 2.99 | NM_005564 |
| Pentraxin-related gene, rapidly induced by IL-1 beta | | |  | | PTX3 | | 2.96 | NM_002852 |
| Defensin, beta 4 | | |  | | DEFB4 | | 2.68 | NM_004942 |
| Complement factor B | | |  | | CFB | | 2.62 | NM_001710 |
| Serum amyloid A2 | | |  | | SAA2 | | 2.31 | NM_030754 |
| Neuronal pentraxin I | | |  | | NPTX1 | | 2.25 | NM_002522 |
| S100 calcium binding protein A9 (calgranulin B) | | |  | | S100A9 | | 2.06 | NM_002965 |
| Serum amyloid A1, transcript variant 1 | | |  | | SAA1 | | 2.02 | NM_000331 |
| S100 calcium binding protein A3 | | |  | | S100A3 | | 1.71 | NM_002960 |
| Small proline-rich protein 2D | | |  | | SPRR2D | | 1.59 | NM_006945 |
|  |  | |  | | |  | |  |
| **Matrix remodeling** | | |  | | |  | |  |
| Plasminogen activator, urokinase | |  | | PLAU | | | 3.44 | NM_002658 |
| Serpin peptidase inhibitor, clade B (ovalbumin), member 2 | |  | | SERPINB2 | | | 2.66 | NM_002575 |
| Serpin peptidase inhibitor, clade B (ovalbumin), member 3 | |  | | SERPINB3 | | | 2.06 | NM_006919 |
| Tenascin C (hexabrachion) | |  | | TNC | | | 1.65 | NM_002160 |
|  |  | | |  | | |  |  |
| **Receptors/ Signal transduction** | | | |  | | |  |  |
| Cytochrome P450, family 1, subfamily A, polypeptide 1 | |  | | CYP1A1 | | | 8.54 | NM_000499 |
| Oxidised low density lipoprotein (lectin-like) receptor 1 | |  | | OLR1 | | | 5.93 | NM_002543 |
| Tumor necrosis factor, alpha-induced protein 2 | |  | | TNFAIP2 | | | 3.87 | NM_006291 |
| Adrenergic, beta-2-, receptor, surface | |  | | ADRB2 | | | 3.25 | NM_000024 |
| Sterile alpha motif domain containing 4A | |  | | SAMD4A | | | 2.60 | NM_015589 |
| Major facilitator superfamily domain containing 2 | |  | | MFSD2 | | | 2.37 | NM_032793 |
| Mitogen-activated protein kinase kinase kinase 8 | |  | | MAP3K8 | | | 2.07 | NM_005204 |
| TNFAIP3 interacting protein 1 | |  | | TNIP1 | | | 2.03 | NM_006058 |
| Suppressor of cytokine signaling 1 | |  | | SOCS1 | | | 1.98 | NM_003745 |
| Dual adaptor of phosphotyrosine and 3-phosphoinositides | |  | | DAPP1 | | | 1.95 | NM_014395 |
| v-yes-1 Yamaguchi sarcoma viral related oncogene homolog | |  | | LYN | | | 1.95 | NM_002350 |
| CD83 molecule, transcript variant 1 | |  | | CD83 | | | 1.91 | NM_004233 |
| Natriuretic peptide receptor A/guanylate cyclase A (atrionatriuretic peptide receptor A) | |  | | NPR1 | | | 1.91 | NM_000906 |
| Transmembrane and coiled-coil domain family 3 | |  | | TMCC3 | | | 1.83 | NM_020698 |
|  |  | | |  | | |  |  |
| **Transcription regulation** | | | |  | | |  |  |
| Zinc finger CCCH-type containing 12A | |  | | ZC3H12A | | | 5.13 | NM_025079 |
| Testis nuclear RNA-binding protein-like | |  | | LOC161931 | | | 2.63 | NM_139174 |
| Nuclear factor of kappa light polypeptide gene enhancer in B-cells 1 (p105) | |  | | NFKB1 | | | 2.61 | NM_003998 |
| Ets homologous factor | |  | | EHF | | | 2.39 | NM_012153 |
| Dilute suppressor | |  | | DSU | | | 2.12 | NM_018000 |
| Human immunodeficiency virus type I enhancer binding protein 2 | |  | | HIVEP2 | | | 2.06 | NM_006734 |
| Promyelocytic leukemia, transcript variant 5 | |  | | PML | | | 2.05 | NM_033244 |
| cAMP responsive element binding protein 5, transcript variant 1 | |  | | CREB5 | | | 1.88 | NM_182898 |
| Cytoplasmic polyadenylation element binding protein 1 | |  | | CPEB1 | | | 1.56 | NM_030594 |
|  | |  | |  | | |  |  |
| **Cytoskeleton/ Cell communication** | | | |  | | |  |  |
| Keratin 6B | |  | | KRT6B | | | 5.95 | NM_005555 |
| Integrin, beta 8 | |  | | ITGB8 | | | 1.76 | NM_002214 |
| Ankyrin repeat domain 44 | |  | | LOC91526 | | | 1.66 | AF086041 |
|  |  | | |  | | |  |  |
| **Transport/ Ion Transport** | | | |  | | |  |  |
| Potassium inwardly-rectifying channel, subfamily J, member 15, transcript variant 1 | |  | | KCNJ15 | | | 4.01 | NM_170736 |
| Solute carrier family 28 (sodium-coupled nucleoside transporter), member 3 | |  | | SLC28A3 | | | 3.29 | NM_022127 |
| TCDD-inducible poly(ADP-ribose) polymerase | |  | | TIPARP | | | 2.54 | NM_015508 |
| Rh family, C glycoprotein | |  | | RHCG | | | 2.48 | NM_016321 |
| Solute carrier organic anion transporter family, member 3A1 | |  | | SLCO3A1 | | | 2.11 | NM_013272 |
| Zinc finger, BED-type containing 2 | |  | | ZBED2 | | | 2.09 | NM_024508 |
| Metallothionein 1M | |  | | MT1M | | | 1.91 | NM_176870 |
| ATPase, H+ transporting, lysosomal 42kDa, V1 subunit C2, transcript variant 2 | |  | | ATP6V1C2 | | | 1.77 | NM_144583 |
| Solute carrier family 2 (facilitated glucose transporter), member 6 | |  | | SLC2A6 | | | 1.55 | NM_017585 |
|  |  | | |  | | |  |  |
| **Cell cycle/ Proliferation** | | | |  | | |  |  |
| Sphingosine-1-phosphate phosphotase 2 | |  | | SGPP2 | | | 2.54 | AK096323 |
| Endothelial differentiation, sphingolipid G-protein-coupled receptor, 3 | |  | | C9orf47 | | | 1.57 | AA534817 |
|  |  | | |  | | |  |  |
| **Metabolism** | | | |  | | |  |  |
| GTP cyclohydrolase 1 (dopa-responsive dystonia), transcript variant 1 | |  | | GCH1 | | | 2.11 | NM_000161 |
| Vanin 1 | |  | | VNN1 | | | 1.99 | NM_004666 |
| Maltase-glucoamylase (alpha-glucosidase) | |  | | MGAM | | | 1.66 | NM_004668 |
