# Supplementary material for: New Microbicidal Functions of Tracheal Glands: Defective Anti-Infectious Response to Pseudomonas aeruginosa in Cystic Fibrosis
Source: PLoS One. 2009 Apr 28;4(4):e5357. doi: 10.1371/journal.pone.0005357 (PMC2670521; doi:10.1371/journal.pone.0005357)
Supplement: Table S6 — Functional classification of down-regulated genes in P. aeruginosa-stimulated CF-TG cells (0.07 MB DOC) [file pone.0005357.s006.doc]

**Table S6.** Functional classification of down-regulated genes in *P. aeruginosa*-stimulated CF-TG cells

| **Category** | **Gene name** | | | | **Symbol** | **Fold Change** | **Accession No.** |
| --- | --- | --- | --- | --- | --- | --- | --- |
| **Chemokines/ Cytokines/ Growth factors** | | | | |  |  |  |
| Fibroblast growth factor 9 (glia-activating factor) | |  | | | FGF9 | -1,75 | NM_002010 |
|  | |  | | |  |  |  |
| **Receptor/ Signal transduction** | | | | |  |  |  |
| DEP domain containing 7 | |  | | | DEPDC7 | -2,81 | NM_139160 |
| Mitogen-activated protein kinase kinase 1 | |  | | | MAP2K1 | -1,96 | BC066984 |
| Mitogen-activated protein kinase 4 | |  | | | MAPK4 | -1,86 | X59727 |
| Endothelin 2 | |  | | | EDN2 | -1,54 | NM_001956 |
| Pleckstrin homology domain containing, family A member 6 | |  | | | PLEKHA6 | -1,48 | NM_014935 |
|  |  | | | |  |  |  |
| **Transcription regulation** | | | | |  |  |  |
| Zinc finger and BTB domain containing 26 | |  | | | ZBTB26 | -2,66 | NM_020924 |
| Zinc finger, CCHC domain containing 2 | |  | | | ZCCHC2 | -2,66 | BC006340 |
| Inhibitor of DNA binding 2, dominant negative helix-loop-helix protein | |  | | | ID2 | -2,26 | NM_002166 |
| Inhibitor of DNA binding 4, dominant negative helix-loop-helix protein | |  | | | ID4 | -1,95 | NM_001546 |
| 2'-5'-oligoadenylate synthetase-like, transcript variant 1 | |  | | | OASL | -1,93 | NM_003733 |
| B-cell CLL/lymphoma 6 (zinc finger protein 51), transcript variant 2 | |  | | | BCL6 | -1,67 | NM_138931 |
| DNA-damage-inducible transcript 4 | |  | | | DDIT4 | -1,66 | NM_019058 |
| HIV-1 Rev binding protein-like | |  | | | HRBL | -1,46 | NM_006076 |
|  | |  | | |  |  |  |
| **Adhesion** | |  | | |  |  |  |
| Platelet/endothelial cell adhesion molecule (CD31 antigen) | |  | | | PECAM1 | -2,61 | NM_000442 |
|  | |  | | |  |  |  |
| **Cytoskeleton/ Cell communication** | | | | |  |  |  |
| Regulating synaptic membrane exocytosis 4 | | |  | | RIMS4 | -4,17 | BC082981 |
| Ankyrin repeat domain 38 | | |  | | ANKRD38 | -2,92 | NM_181712 |
| PDZ domain containing 1 | | |  | | PDZK1 | -1,84 | NM_002614 |
| Gap junction protein, alpha 5, 40kDa (connexin 40), transcript variant A | | |  | | GJA5 | -1,78 | NM_005266 |
| LINE-1 type transposase domain containing 1 | | |  | | L1TD1 | -1,60 | NM_019079 |
| Leucine rich repeat and fibronectin type III domain containing 3 | |  | | | LRFN3 | -1,21 | NM_024509 |
|  |  | | | |  |  |  |
| **Transport/ Ion Transport** | | | | |  |  |  |
| Potassium channel tetramerisation domain containing 11 | | |  | | KCTD11 | -1,64 | NM_001002914 |
| HSPC049 protein | | |  | | HSPC049 | -1,43 | NM_014149 |
|  | | |  | |  |  |  |
| **Cell cycle/ Proliferation** | | | | |  |  |  |
| Similar to TSG118.1 | | |  | | LOC400506 | -1,49 | CR625565 |
| Rho guanine nucleotide exchange factor (GEF) 17 | | |  | | ARHGEF17 | -1,46 | NM_014786 |
|  |  | | | |  |  |  |
| **Apoptosis** |  | | | |  |  |  |
| Caspase 9, apoptosis-related cysteine peptidase, transcript variant alpha | | |  | | CASP9 | -1,80 | NM_001229 |
| Death inducer-obliterator 1, transcript variant 1 | | |  | | DIDO1 | -2,52 | NM_022105 |
|  |  | | | |  |  |  |
| **Metabolism** |  | | | |  |  |  |
| Serine palmitoyltransferase, long chain base subunit 2-like (aminotransferase 2) | | | |  | SPTLC2L | -1,61 | AK075271 |
| Helicase (DNA) B | | | |  | HELB | -1,64 | NM_033647 |
|  |  | | | |  |  |  |
| **Protein degradation** | | | | |  |  |  |
| F-box and leucine-rich repeat protein 2 | | | |  | FBXL2 | -1,76 | NM_012157 |
