# Supplementary material for: New Microbicidal Functions of Tracheal Glands: Defective Anti-Infectious Response to Pseudomonas aeruginosa in Cystic Fibrosis
Source: PLoS One. 2009 Apr 28;4(4):e5357. doi: 10.1371/journal.pone.0005357 (PMC2670521; doi:10.1371/journal.pone.0005357)
Supplement: Table S7 — Primers used for qRT-PCR (0.04 MB DOC) [file pone.0005357.s007.doc]

**Table S7.** Primers used for qRT-PCR

| Gene | Forward primer | Reverse primer |
| --- | --- | --- |
| IL-1α | 5'-gtaagctatggcccactcca-3' | 5'-gcctccaggtcatcatcagt-3' |
| IL-1β | 5'-gctgaggaagatgctggttc-3' | 5'-tccatatcctgtccctggag-3' |
| IL-32 | 5'-gtggcggcttattatgagga-3' | 5'-cctggaaccatctcatgacc-3' |
| TNFSF14 | 5'-ctggcgtctaggagagatgg-3' | 5'-cccagctgagtctcccataa-3' |
| LIF | 5'-tgccaatgccctctttattc-3' | 5'-aggtgccaaggtacacgact-3' |
| CXCL1 | 5'-agggaattcaccccaagaac-3' | 5'-taactatgggggatgcagga-3' |
| PLAU | 5'-tgaggtggaaaacctcatcc-3' | 5'-ggcaggcagatggtctgtat-3' |
| S100A8 | 5'-atgccgtctacagggatgac-3' | 5'-acgcccatctttatcaccag-3' |
| S100A9 | 5'-cagctggaacgcaacataga-3' | 5'-ttgtgtcaggtcctccatga-3' |
| IL-24 | 5'-agggccaagaattccacttt-3' | 5'-gggcactcgtgatgttatcc-3' |
| CXCL10 | 5'-accgtacgctgtacctgcat-3' | 5'-caacacgtggacaaaattgg-3' |
| IFNγR2 | 5'-agtccaggcacaactgcttt-3' | 5'-ccacggagatcaggatgact-3' |
| TLR2 | 5'- gccaaagtcttgattgattgg-3' | 5'-ataccacaggccatggaaac-3' |
| 18S rRNA | 5'-tcaagaacgaaagtcggagg-3' | 5'-cagctttgcaaccatactcc-3' |
